# RAS inhibition limits oncogenic signaling and tumorigenesis in neuroblastoma regardless of RAS mutational status

**DOI:** 10.1038/s41598-026-63488-x

**Published:** 2026-07-31

**Authors:** Mubashir Mintoo, Vinodh Rajagopalan, Rachel Greathouse, Cecile Nasarre, Tarah Trebino, Dianicha Santana, Kevin O. Flores-Galvez, Gayatri Ketavarapu, Akiko Koide, Fiona E. Hood, Ian A. Prior, Mark R. Philips, Shohei Koide, Bill Chiu, John P. O’Bryan

**Affiliations:** 1 Department of Biochemistry and Molecular Biology, Hollings Cancer Center, Medical University of South Carolina, 86 Jonathan Lucas St. MSC 955, Rm HO712E, Charleston, SC 29425 USA; 2 Ralph H. Johnson VA Medical Center, Charleston, SC 29401 USA; 3 Department of Surgery, Division of Pediatric Surgery, Stanford University, Stanford, CA 94305 USA; 4 Department of Pharmacology, University of Illinois Cancer Center, University of Illinois at Chicago, Chicago, IL 60612 USA; 5 Perlmutter Cancer Center, New York University Langone Health, New York, NY 10016 USA; 6 Department of Medicine, New York University School of Medicine, New York, NY 10016 USA; 7 Cellular and Molecular Physiology, Institute of Systems Molecular and Integrative Biology, University of Liverpool, Crown St, Liverpool, L69 3BX UK; 8 Molecular and Clinical Cancer Medicine, Institute of Systems Molecular and Integrative Biology, University of Liverpool, Crown St, Liverpool, L69 3BX UK; 9 Department of Biochemistry and Molecular Pharmacology, New York University School of Medicine, New York, NY 10016 USA

**Keywords:** Neuroblastoma, RAS GTPase, MRTX-1133, Sotorasib, RMC-7977, NS1 monobody, Cancer, Cell biology, Oncology

## Abstract

Neuroblastoma (NBL) is a rare and aggressive pediatric malignancy with a 5-year survival rate of 40–50% for patients diagnosed with high-risk disease. NBLs arise in the sympathetic nervous system and causes 11% of all pediatric cancer deaths. MYCN amplification or overexpression drives 22% of NBL cases while deregulation of genes including c-MYC, ALK, PHOX2B, and LIN28B also plays a critical role in NBL initiation and progression. Although RAS proto-oncogenes are rarely mutated in primary NBL (< 2%), studies suggest that elevated RAS activity may contribute to NBL tumorigenesis and progression. Here, we explore targeted RAS inhibition as a potential therapeutic strategy for NBL. Using mutation-specific inhibitors of KRAS, we demonstrate the importance of mutant KRAS in NBL. Furthermore, using anti-RAS biologics and a pan RAS inhibitor, we also demonstrate the importance of wild type (WT) RAS activity in non-RAS mutant NBLs. Direct RAS inhibition decreases NBL cell proliferation, colony formation, and migration in vitro in NBL lines differing in MYCN and RAS mutation status. Furthermore, inhibition of WT RAS suppresses the growth of specific NBL lines in vivo. Our findings suggest that targeted RAS inhibition, even in the absence of RAS mutation, may represent a promising therapeutic approach for NBL patients.

**Supplementary Information:**

The online version contains supplementary material available at https://doi.org/10.1038/s41598-026-63488-x.

## Introduction

NBL is a neuroendocrine neoplasm originating in the embryonic neural crest, giving rise to neoplastic growths within the adrenal glands and/or sympathetic ganglia^1,2^. NBL is the most common extracranial solid malignancy identified within the first year of life, with 25–50 cases per million individuals^3^. Occurrence differs globally, with NBL more frequent in Caucasian populations than in populations of African ancestry^3^. NBL accounts for approximately 8–10% of pediatric malignancies and 15% of all pediatric cancer-related mortality^2^. At diagnosis, 45% of patients present with high-risk disease, with roughly only half achieving long-term remission despite receiving multiple therapeutic modalities including retinoid therapy, myeloablative chemotherapy, anti-GD2 immunotherapy (dinutuximab), and hematopoietic stem cell transplantation^4^.

NBL exhibits remarkable intratumoral heterogeneity, manifesting a broad spectrum of clinical prognoses ranging from spontaneous tumor regression to highly aggressive and therapy-resistant disease. High-risk NBLs harbor a very low frequency of recurrent somatic mutations^5^. While certain genetic changes including MYCN amplification, 1p deletion, 11q deletion, and 17q gain have been linked to poor prognosis, the specific genetic factors associated with disease relapse remain unidentified^6^. About 20–30% of NBL tumors exhibit amplification of the MYCN proto-oncogene located on chromosome 2p24, a factor correlated with aggressive disease and unfavorable patient survival outcomes^6^. Thus, MYCN amplification has emerged as a robust, stage-independent prognostic marker in NBL^7^.

Recently, there has been a noteworthy push to enhance the classification of patients with NBL based on their age, presence of metastases, genomic alterations, and differentiation state. Roughly 1–2%, of NBL cases originate from a genetic inheritance pattern within families, exhibiting autosomal dominance^8^. A shared genetic link to NBL was first discovered in families where the cancer appeared alongside related neural crest disorders such as Hirschsprung disease^9^. Studying such families with congenital central hypoventilation syndrome led to the identification of mutations in the PHOX2B gene, unveiling its key role in these linked conditions and its contribution to NBL susceptibility^10^.

In addition to MYCN and PHOX2B, the anaplastic lymphoma kinase (ALK) is another crucial oncogene driving NBL development. Overexpression of ALK contributes to tumorigenesis in high-risk NBLs^11,12^ and approximately 5–10% of NBLs harbor activating ALK mutations^13^. Genetic knockdown of ALK results in growth inhibition of both wild type (WT) and mutant ALK cell lines^14^. Although Crizotinib, a first generation ALK inhibitor, has limited clinical efficacy^15^, newer generation ALK inhibitors show improved activity in ALK-mutant NBLs but induce complete responses in only a small fraction of NBL patients^16–18^. Other factors that contribute to NBL tumorigenesis include loss of heterozygosity at chromosome 14q, CDKN2A, loss of NF1, amplification of DDX1 and MDM2, neurotrophin receptor overexpression, aberrant expression of GD2 ganglioside, polycomb protein Bmi-1, misregulated microRNA expression (e.g., miR-10b, miR-29a/b, miR-335) and mutations in ATRX, CHEK2 and BARD1^5,19–23^.

Despite implicating various pathways in NBL tumorigenesis, targeting any single pathway has not translated to substantial improvements in disease-free survival. This stark reality is exemplified by the NCI-supported TARGET initiative, which yielded no frequent (> 10%) somatic mutations driving NBL development^5^. These findings hint at yet-to-be-discovered mechanisms driving NBL development and progression. One promising avenue of investigation lies in the potential role of the RAS pathway in NBL. Although RAS mutations are rare in NBLs (< 2%)^24^, recent findings reveal their frequent presence in relapsed and refractory cases^25^. In addition, dysregulation of RAS regulatory proteins such as receptor tyrosine kinases (RTKs), NF1, PTPN11, and GAB2 may contribute to RAS pathway activation in NBL^26–28^. Hence, increased RAS activity, even in the absence of RAS mutations, may contribute to NBL tumorigenesis.

To test the importance of RAS in NBL tumor biology, we have utilized mutation-specific KRAS inhibitors as well as inhibitors of WT RAS^29,30^. We found that NBLs with oncogenic *KRAS* mutations exhibit a dependency on mutant RAS. Furthermore, a subset of NBLs lacking *RAS* mutations is unexpectedly dependent on WT RAS activity. These findings suggest that targeted inhibition of RAS, even in the absence of RAS mutations, may provide a new therapeutic approach for a subset of NBL patients.

## Materials and methods

### Cell lines and chemicals

The NBL lines used in this study are described in Supplementary Table 1. CHP-134 (RRID: CVCL_1124), IMR-5 (RRID: CVCL_1306), SK-N-AS (RRID: CVCL_1700), have been previously described^31^ and were provided by Naohiko Ikegaki (University of Illinois at Chicago). KELLY was obtained from Sigma (CAT# 92110411, RRID: CVCL_2092). LA-N-6 (RRID: CVCL_1363), SK-N-FI (RRID: CVCL_1702) and NB-EBc1 (RRID: CVCL_E218) cells were obtained from the Children’s Oncology Group Cell Culture/Xenograft Repository (www.cccells.org). SH-EP (RRID: CVCL_0524) cells were kindly provided by Michael Hogarty (Children’s Hospital of Philadelphia), and IMR-32 (RRID: CVCL_0346) and SH-SY5Y (RRID: CVCL_0019) cells provided by Bernard Weissman (University of North Carolina at Chapel Hill). RASless HEK 293 cells were provided by Frank McCormick and have been previously described^32^. Cell authenticity was periodically confirmed by analysis of STR. NBL cell lines were maintained in RPMI with 10% fetal bovine serum at 37 °C in 5% CO_2_ except NB-EBc-1 which was maintained in Iscove Modified Dulbecco medium (IMDM) (ATCC #30-2005) supplemented with ITS (5 µg/mL insulin, 5 µg/mL transferrin, 5 ng/mL selenous acid; ThermoFisher #41400045). hEGF (Sigma #E9644), Puromycin (Gibco #A1113803) was used at 1 µg/ml, G418 (GIBCO #11811-031) at 200 µg/ml, and Doxycycline (DOX) (Sigma #D9891) at various concentrations as indicated. Mycoplasma testing was performed every 6 months with Lonza MycoAlert Detection kit (#LT07-218). Following are the specific and panRAS inhibitors used in this study: MRTX-1133 (Chemietek #CT-MRTX1133)^33^, Sotorasib (#S8830, Selleckchem, Houston, TX)^34^, RMC-7977 (Chemietek # CT-RMC7977) (Supplementary Fig. 1A)^30^.

### Antibodies and siRNA

The following antibodies were used: phospho-ERK (Thr202/Tyr204, CST #9101, RRID: AB_331646), total ERK (CST #9102, RRID: AB_330744), KRAS (Millipore Sigma #WH0003845M1-100UG, RRID: AB_1842235), HRAS (Proteintech #18295-1-AP, RRID: AB_2121046), NRAS (Abcam #AB300431, RRID: AB_3662857), β-tubulin (Sigma #T4026, RRID: AB_477577), monoclonal FLAG (Clone M2, Millipore Sigma #F1804, F7425, RRID: AB_262044), Vinculin (Santa Cruz #sc-25336, RRID: AB_628438), secondary anti-mouse (Biorad #1706516, RRID: AB_11125547) and secondary anti-rabbit (Biorad #1706515, RRID: AB_11125142). The following siRNAs and transfection reagents were used in the study: siRNA (Thermo Fisher KRAS - s7940, HRAS - s806, NRAS - s54); scramble control siRNA (Dharmacon #D-001810-10-20); Lipofectamine Transfection Reagent (Thermo Fisher # 13778150).

### DNA constructs and lentivirus generation

pCW5.1-CFP-FLAG-NS1 plasmid used in the study was described previously^35^. Lentivirus was generated using the pCW5.1-CFP-FLAG-NS1 monobody construct by the Viral Vector Core (Director, Negin Martin), National Institute of Environmental Health Sciences, NIH, Research Triangle Park, NC, USA as described previously^29,35^.

### Immunoblotting

Parental NBL cells were seeded in 100 mm petri plates then incubated with inhibitors at the indicated concentrations and for the indicated times. DMSO (0.1%) was used as a vehicle control. For analysis of NS1 effects, cells were treated with doxycycline (DOX) at indicated concentrations for 48 h to induce NS1 expression. Ethanol (0.05%) was used as vehicle control. Cells were then washed with PBS and lysed with PLC-LB buffer containing (50 mM HEPES, pH 7.5, 150 mM NaCl, 10% glycerol, 1% Triton X-100, 1 mM EGTA, 1.5 mM magnesium chloride, 100 mM sodium fluoride supplemented with 1 mM sodium orthovanadate, 10 mg/ml leupeptin, and 10 mg/ml aprotinin, Benzamidine HCl). Lysates were incubated at 4 °C with gentle rocking for 1 h then centrifuged at 14,000 RPM at 4 °C for 15 min. The supernatants were transferred to fresh tubes and protein concentrations were determined by Pierce BCA Protein Assay kit (Thermo Fisher #PI23225).

### Proliferation assay

NBL cells (1000 per well) were plated in 96-well plates in complete media and treated with inhibitors or DOX for the indicated number of days. Media was then removed and replaced with 100 µl serum-free media to which 100 µl of CellTiterGlo reagent (Promega, Madison, WI, USA; #G7571) was added. Luminescence was quantified on a CLARIOStar (BMG LABTECH) 96-well microtiter plate luminometer according to the manufacturer’s instructions. Alternatively, proliferation was measured using Calcein AM dye (ThermoFisher #C1430). Briefly, cells were seeded in triplicate wells of a 96-well plate for each treatment condition. On Day 1, inhibitors were added and refreshed every 48 h until Day 5. Proliferation was measured using Calcein AM dye on Day 1 (prior to treatment) and again on Day 5. Calcein AM was applied to the designated wells and incubated for 15 min. Following incubation, live-cell counts were obtained using the ImageXpress Pico Automated Cell Imaging System, Cell Reporter Xpress software, version 2.9.4.19394 (Molecular Devices, LLC; www.moleculardevices.com/products/cellular-imaging-systems/high-content-imaging/imagexpress-pico?)

### Cell signaling assays

For determining ERK activation, cell lysates were harvested at the indicated times following treatment with monobody or inhibitors. ERK and pERK levels were analyzed via Western Blot using α-ERK (Cell Signaling Technology) and α-pERK (Cell Signaling Technology) antibodies as previously described^36^. ERK and pERK protein levels were quantified using ImageJ software and normalized to an internal control (vinculin). pERK/ERK ratios were determined for each experiment and normalized to control untreated sample where indicated. RAS-GTP levels were measured using the GST-RAF RBD pull down assays as previously described^37^. Briefly, GST-RAF RBD was expressed and purified from bacteria in buffer containing 25 mM HEPES (pH 7.5), 150 mM NaCl, 10% glycerol, 1% Triton X-100, 25 mM MgCl_2_, 1 mM EDTA, 25 mM NaF, 0.25% DOC, 1 mM DTT, along with protease and phosphatase inhibitors. Cell lysates prepared from NBL cell lines were used as a source of RAS and incubated with bacterially purified GST-RAF RBD (immobilized on Glutathione Sepharose beads) in 25 mM HEPES, pH 7.5, 10% glycerol, 150 nM NaCl, 1%TritionX-100, 0.25% sodium deoxycholate, 25 mM MgCl_2_, 1 mM EDTA for 1 h at 4 °C. Beads were then washed 4 times with PLC buffer and bound proteins analyzed by Western blot. Extent of RAS co-precipitated with GST-RBD was quantified by densitometry using NIH ImageJ.

### Soft agar assay

Anchorage-independent growth was assessed as previously described^38^. Plates were incubated at 37 °C for 2–3 weeks with media changes 1–2 times per week. DOX (4 µg), MRTX-1133/Sotorasib (50 nM), RMC-7977 (100 nM), or vehicle control was added to wells containing NS1-expressing or parental NBL cells, respectively. Colony staining was performed with 200 µl of MTT (3-(4,5-dimethylthiazol-2-yl)-2,5-diphenyltetrazolium bromide) prepared at a concentration of 2 mg/ml in PBS and quantification was done using ImageJ software. Threshold and colony size cutoff parameters were determined by evaluating representative control and treatment images and selecting settings that accurately identified visually distinguishable colonies while excluding debris and staining artifacts. The same parameters were subsequently applied to all images for unbiased quantification as described here^39^. Colony number per well represents the average of three wells ± SD.

### Scratch assay

NBL cells were seeded in 6-well plates and allowed to form a confluent monolayer. A scratch, mimicking a wound, was then made using a sterile pipette tip. Following a thorough wash with PBS to remove detached cells, fresh media was added and an initial image (“zero day”) was captured. The NBL cells were treated with RAS inhibitors (MRTX-1133, Sotorasib, or RMC-7977), DOX (to induced NS1) or vehicle control (0.1% DMSO for inhibitors; 0.05% ethanol for NS1). ImageJ version 1.54 g (NIH; www.wsr.imagej.net/ij/download.html) was used for the quantification of final scratch as compared to initial scratch area. % wound closure was analyzed with formula given below.

$$\:\mathrm{\%}\:\mathrm{W}\mathrm{o}\mathrm{u}\mathrm{n}\mathrm{d}\:\mathrm{c}\mathrm{l}\mathrm{o}\mathrm{s}\mathrm{u}\mathrm{r}\mathrm{e}=\frac{\mathrm{I}\mathrm{n}\mathrm{i}\mathrm{t}\mathrm{i}\mathrm{a}\mathrm{l}\:\mathrm{s}\mathrm{c}\mathrm{r}\mathrm{a}\mathrm{t}\mathrm{c}\mathrm{h}\:\mathrm{a}\mathrm{r}\mathrm{e}\mathrm{a}\:-\:\mathrm{F}\mathrm{i}\mathrm{n}\mathrm{a}\mathrm{l}\:\mathrm{s}\mathrm{c}\mathrm{r}\mathrm{a}\mathrm{t}\mathrm{c}\mathrm{h}\:\mathrm{a}\mathrm{r}\mathrm{e}\mathrm{a}}{\mathrm{I}\mathrm{n}\mathrm{i}\mathrm{t}\mathrm{i}\mathrm{a}\mathrm{l}\:\mathrm{s}\mathrm{c}\mathrm{r}\mathrm{a}\mathrm{t}\mathrm{c}\mathrm{h}\:\mathrm{a}\mathrm{r}\mathrm{e}\mathrm{a}}\:,\:100$$

### Quantification of RAS isoform abundance by mass spectrometry

RAS protein isoform quantitation was performed as described previously^40,41^. Briefly, 6–12 million cells were lysed using lysis buffer (50 mM Tris, pH 7.5, 150 mM NaCl, 1% NP40 substitute, 1/250 mammalian protease inhibitor cocktail [Sigma]) and protein concentration quantified using BCA assay (Fisher #PI23228). Heavy-labelled protein standards (2 ng His-KRAS4B, 1 ng His-KRAS4A, 1 ng His-HRAS and 1 ng His-NRAS) were spiked into 20 µg cell lysate and proteins separated by SDS-PAGE. Proteins were reduced, alkylated and digested in-gel using 5 ng/µl Trypsin Gold (Promega). Extracted peptides were desalted using C18 before analysis on a QTrap 6500 (Sciex) with an in-line Dionex U3000 nano-LC system (Thermo) mounted with a NanoAcquity 5 μm particle size, 180 μm 20 mm C18 trap and 1.7 μm particle size, 75 μm 100 mm C18 analytical column (Waters). Columns were maintained at 40 °C. A gradient of 2 − 50% acetonitrile/0.1% formic acid (v/v) over 45 min was applied to the columns at a flow rate of 300 nL/min. The NanoSpray III source of the mass spectrometer was fitted with a 10 μm inner diameter PicoTip emitter (New Objective). The mass spectrometer was operated in positive ion mode using Analyst TF Software, version 1.6 (SCIEX, Framingham, MA, USA; www.sciex.com) and the MIDAS approach (MRM-initiated detection and sequencing) was used to quantify and confirm the identity of the analytes of interest. Area under curve was extracted for at least 3 combined transitions in Analyst TF Software, version 1.6 (SCIEX, Framingham, MA, USA; www.sciex.com), and further analysis performed Microsoft Excel version 16.101.2 for Mac (Microsoft Corporation, Redmond, WA, USA; www.microsoft.com) and GraphPad Prism version 10.6.1 for Mac (GraphPad Software, Boston, Massachusetts USA; www.graphpad.com). For RAS mutant cell lines, one sample run measures all 4 wildtype RAS isoforms, while a separate sample run measures the mutant isoform. For Supplementary Fig. 6D, mean KRAS4B (*n* = 2 biological repeats) and a single KRAS4A repeat are shown as a proportion of their sum. Two biological repeats for KRAS4B were performed for each cell line; however, KRAS4A measurements were only from a single sample read due to the inability to detect peptides for the other sample. Standard error for KRAS4B was calculated in Excel. For Supplementary Fig. 6E, total RAS was calculated from summing PanRAS (wildtype) and PanRAS (mutant) peptide reads (PanRAS peptide comprises RAS residues 6–16, thus including the G12 and G13 codon mutation sites). HRAS and NRAS reads were subtracted from this to yield total KRAS as the KRAS4A peptide detection was unreliable. Error bars represent standard error from *n* = 2 biological repeats.

### Determining specificity of NS1 to KRAS isoforms

KRAS4B (residues 1-174) with N-terminal His_6_ and Avi-tags followed by a TEV cleavage site was produced in *Escherichia coli* BL21(DE3) expressing BirA for in-cell biotinylation, as described^35^. KRAS4A (residues 1-174) was produced essentially the same manner. Binding of NS1 monobody displayed on the yeast cell surface to KRAS4A and KRAS4B was measured, as described^29,35^. The molecular graphics (Supplementary Fig. 6B) were produced using PyMOL 3.1.0 (Schrödinger; www.pymol.org/).

### Orthotopic tumor model

All animal studies were performed under the approval of the Institutional Animal Care and Use Committees (IACUC) and Institutional Biosafety Committees (IBC) at Stanford University. All methods were performed in accordance with the relevant guidelines, regulations and protocols approved by the Stanford IACUC and IBC. Approximately 1 × 10^6^ cells for each stable NBL line were collected in phosphate-buffered saline (PBS) an injected into the left adrenal gland of athymic nude mice (Charles River, Wilmington, MA) through a laparotomy as described^42–44^. Tumor formation was followed weekly by ultrasound measurements. NS1 expression was induced by adding DOX to the drinking water as previously described^38^. RMC-7977 was administered by daily oral gavage (10 mg/kg) 5x per week once tumors were detected by ultrasound. Animals were euthanized at the experimental endpoint (when the tumor volume exceeded 1000 mm^3^) by CO_2_ asphyxiation from an IACUC approved source. Tumor volumes between treatment groups were analyzed using ANOVA and Kaplan Meier survival curves were plotted and statistically compared using Log rank (Mantel-Cox) test using GraphPad Prism version 11.0.2 (100) (GraphPad Software, Boston, Massachusetts USA; www.graphpad.com).

## Results

**Fig. 1:**
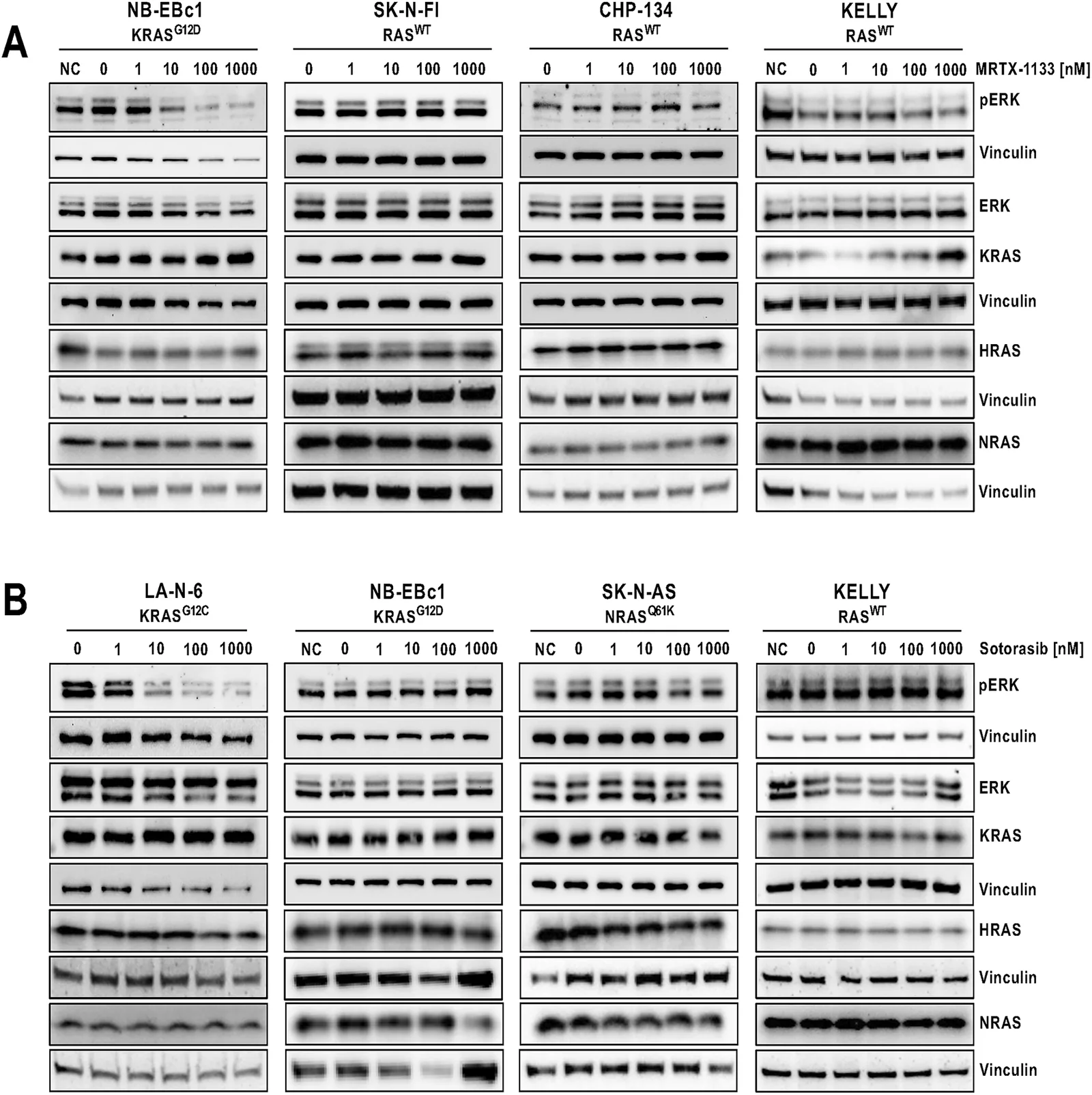
Mutation-specific KRAS inhibitors reduce ERK activation in KRAS mutant NBLs. **(A)** NBL cell lines were treated with the indicated concentrations of MRTX-1133 for 48 h. **(B)** Same as in **(A)** except the indicated cells were treated with Sotorasib. NC, negative control lacking both DMSO and inhibitors.

Dysregulation of RAS signaling pathways is frequently observed in various cancers and linked to tumorigenesis and aggressive disease progression. While RAS mutations are rare in NBLs (see Supplementary Table 2), several established NBL cell lines harbor mutant RAS alleles, although their importance to the transformed phenotype has not been addressed. We observed significant heterogeneity in ERK activation, RAS isoform expression and RAS GTP levels, across NBL cell lines with and without oncogenic RAS (Supplementary Figs. 1B-C). Given the recent development of mutation-selective KRAS inhibitors (e.g., MRTX-1133 and Sotorasib)^33,34^, we tested the importance of oncogenic KRAS in mediating the tumorigenic properties of these NBLs. Treatment with MRTX-1133, a selective KRAS^G12D^ inhibitor^33^, resulted in a dose-dependent decrease in ERK activation of KRAS^G12D^-mutant NB-EBc1 cells but did not affect non-KRAS^G12D^ mutant NBLs (Figs. 1A, Supplementary Fig. 2A & 3A). Similarly, treatment with Sotorasib, a selective KRAS^G12C^ inhibitor^34^, resulted in a dose-dependent decrease in ERK activation of KRAS^G12C^-mutant LA-N-6 cells but did not affect non-KRAS^G12C^ mutant NBLs (Fig. 1B, Supplementary Fig. 2B & 3B).

**Fig. 2:**
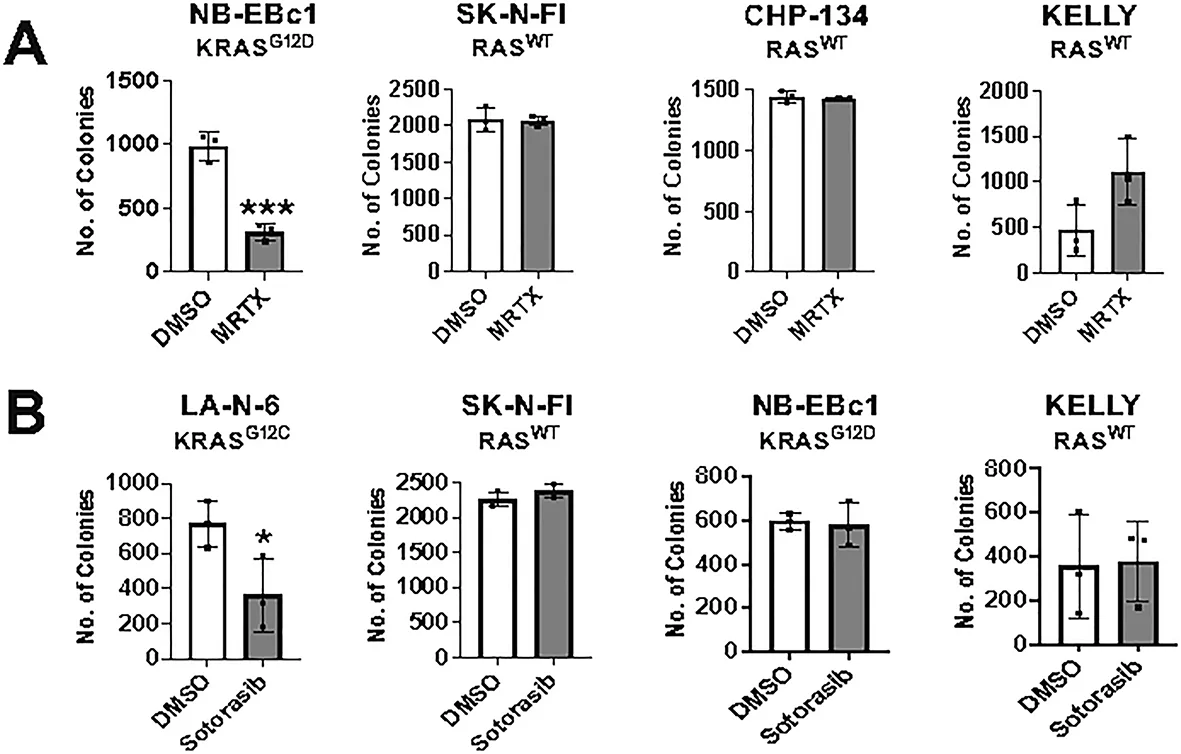
KRAS inhibition reduces anchorage-independent growth of KRAS mutant NBLs. NBL cells were plated in soft agar in the presence of DMSO, MRTX-1133 (50 nM) **(A)** or Sotorasib (50 nM) **(B)** and allowed to grow for 3 to 4 weeks. Graphs represent the average colony number from three independent wells ± SD. The experiments were done in three technical replicates for each cell line. Asterisks represent p values determined by comparison of colony numbers between DMSO and MRTX/Sotorasib conditions using a Student’s t-test. *, *p* < 0.05, **, *p* < 0.01, ***, *p* < 0.001; samples without asterisks were not statistically different.

To further examine the importance of oncogenic RAS in NBL tumor biology, we assessed the effect of these mutation-specific KRAS inhibitors on anchorage-independent growth of NBLs. MRTX-1133 significantly inhibited colony formation in NB-EBc1 cells but did not affect non-KRAS^G12D^ mutant NBLs (Figs. 2A and Supplementary Fig. 4A). Similarly, Sotorasib reduced colony formation of LA-N-6 cells without affecting non-KRAS^G12C^ mutant NBLs (Figs. 2B and Supplementary Fig. 4B). MRTX-1133 similarly inhibited the proliferation and migration of NB-EBc1 (Supplementary Figs. 4C-D) and Sotorasib inhibited migration of LA-N-6 cells (Supplementary Fig. 4D). Together, these data indicate that oncogenic KRAS drives pro-proliferative signaling, migration, and anchorage-independent growth in KRAS-mutant NBLs.

Given the prominent role of oncogenic RAS in driving tumorigenic signaling in a subset of NBLs, we next explored whether WT RAS contributes to the oncogenic properties of NBLs lacking RAS mutations. In contrast to the rarity of RAS mutations in NBL (Supplementary Table 2), alterations in upstream regulators of RAS such as ALK, PTPN11, and NF1 occur in ~ 15% of NBLs and may lead to aberrant RAS activation and signaling^5,12,23,45^. These observations suggest that WT RAS may play a critical role in sustaining oncogenic signaling, even in the absence of RAS mutations, by acting as a central node in the MAPK and PI3K pathways, both of which are implicated in NBL proliferation and survival. Indeed, RAS GTP levels are elevated in a subset of NBLs lacking RAS mutations, e.g., SK-N-FI and CHP-134 (Supplementary Fig. 1C).

**Fig. 3:**
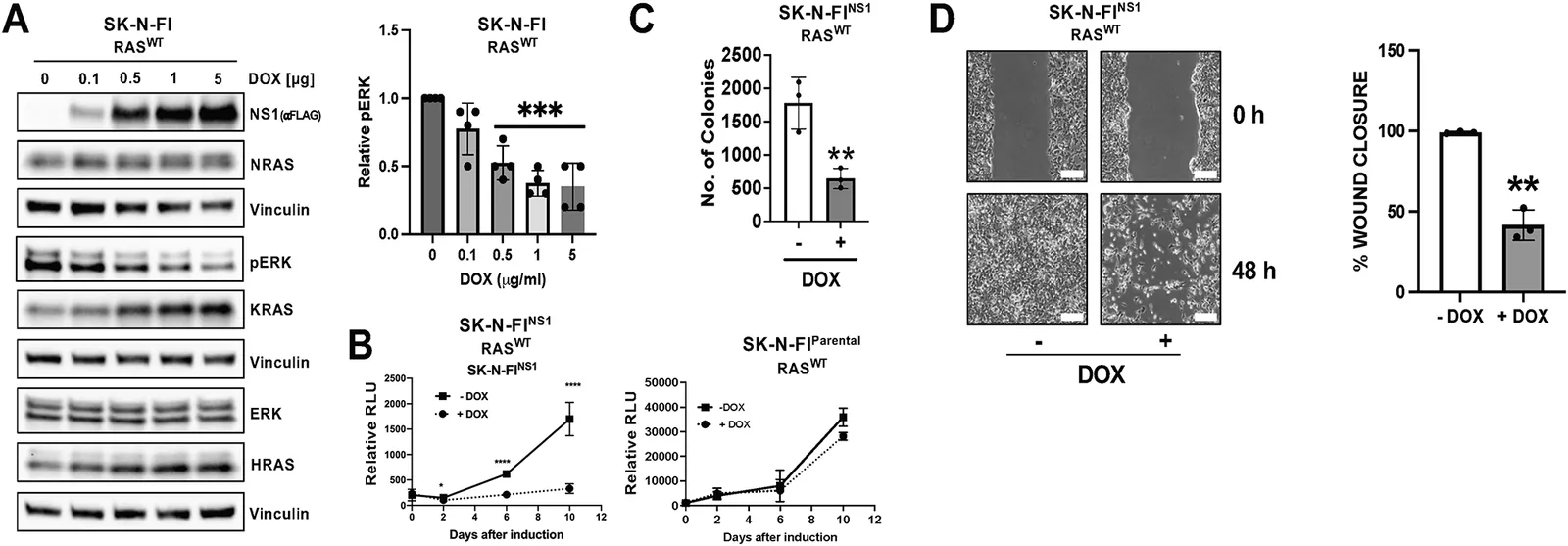
NS1 monobody inhibits SK-N-FI cells. **(A)** ERK activation was measured as in Fig. 1A. **(B)** DOX-induced expression of NS1 in SK-N-FI^NS1^ reduced proliferation. Parental cells treated with DOX were unaffected. Error bars represent standard deviation of three independent wells per time point. **(C)** NS1 induction reduces SK-N-FI anchorage-independent growth. Results from 3 technical replicates were quantified as in Fig. 2A and B. **, *p* < 0.01. **(D)** NS1 induction reduces the migration of SK-N-FI cells following wounding. Results are the average from 3 independent experiments White scale bars, 500 μm. **, *p* < 0.01.

To address the importance of WT RAS in NBL, we utilized the tool biologic NS1 monobody which inhibits HRAS and KRAS, but not NRAS, by disrupting RAS nanoclustering thereby inhibiting the ability of RAS to activate downstream effectors such as RAF and PI3K^29,46^. Expression of NS1 suppressed ERK activation in SK-N-FI (Fig. 3A and Supplementary Fig. 5A). This reduction in pERK levels mirrored the observations with MRTX-1133 and Sotorasib in RAS-mutant cells, highlighting the importance of WT H/KRAS-mediated signaling in SK-N-FI cells. In contrast, pERK levels in KELLY and IMR-5 cells were unaffected by NS1 expression suggesting that these NBL lines were refractory to inhibition of WT KRAS or HRAS (Supplementary Fig. 5A & B). NB-EBc1 cells did not express enough NS1 to inhibit RAS mediated ERK activation (Supplementary Fig. 5A). SK-N-AS and SH-EP cells both harbor mutant NRAS alleles and were unaffected by NS1 expression, as expected^29,46^(Supplementary Figs. 5A). However, expression of Mb24, an NRAS selective monobody biologic, reduced pERK levels in SH-EP cells demonstrating their reliance on oncogenic NRAS^36^. In addition to these effects on ERK, NS1 reduced cell proliferation, anchorage-independent growth, and migration of SK-N-FI cells (Figs. 3B-D). Although a similar reduction in cell proliferation was observed in CHP-134^NS1^ cells which are WT for RAS, this effect was not attributable to NS1-mediated inhibition of RAS signaling but rather to non-specific inhibitory effects of DOX as observed in parental CHP-134 cells (Supplementary Fig. 5C).

The *KRAS* locus encodes two distinct proteins, KRAS4A and KRAS4B, arising from differential splicing of the 4th coding exon^47^. Our previous work with NS1 used the KRAS4B protein which is the more widely expressed KRAS isoform^29^. Here, we discovered that NS1 does not bind KRAS4A, likely due to a steric clash caused by R151 in KRAS4A that replaces Gly in KRAS4B (Supplementary Figs. 6A-C). These results raised the possibility that the inability of NS1 to inhibit certain NBL lines may be due to signaling driven by KRAS4A or NRAS, the two isoforms to which NS1 does not bind. Thus, we determined the levels of individual RAS protein isoforms by quantitative mass spectrometry^40,48^. KRAS4B constituted greater than 90% of the total KRAS protein in the NBL lines analyzed (Supplementary Fig. 6D). Furthermore, several lines expressed abundant NRAS whereas HRAS represented less than 20% of the total RAS protein in NBL cells (Supplementary Fig. 6E). These results suggest that the inability of NS1 to inhibit NBL lines was not due to expression of KRAS4A but may be due in part to NRAS expression.

**Fig. 4:**
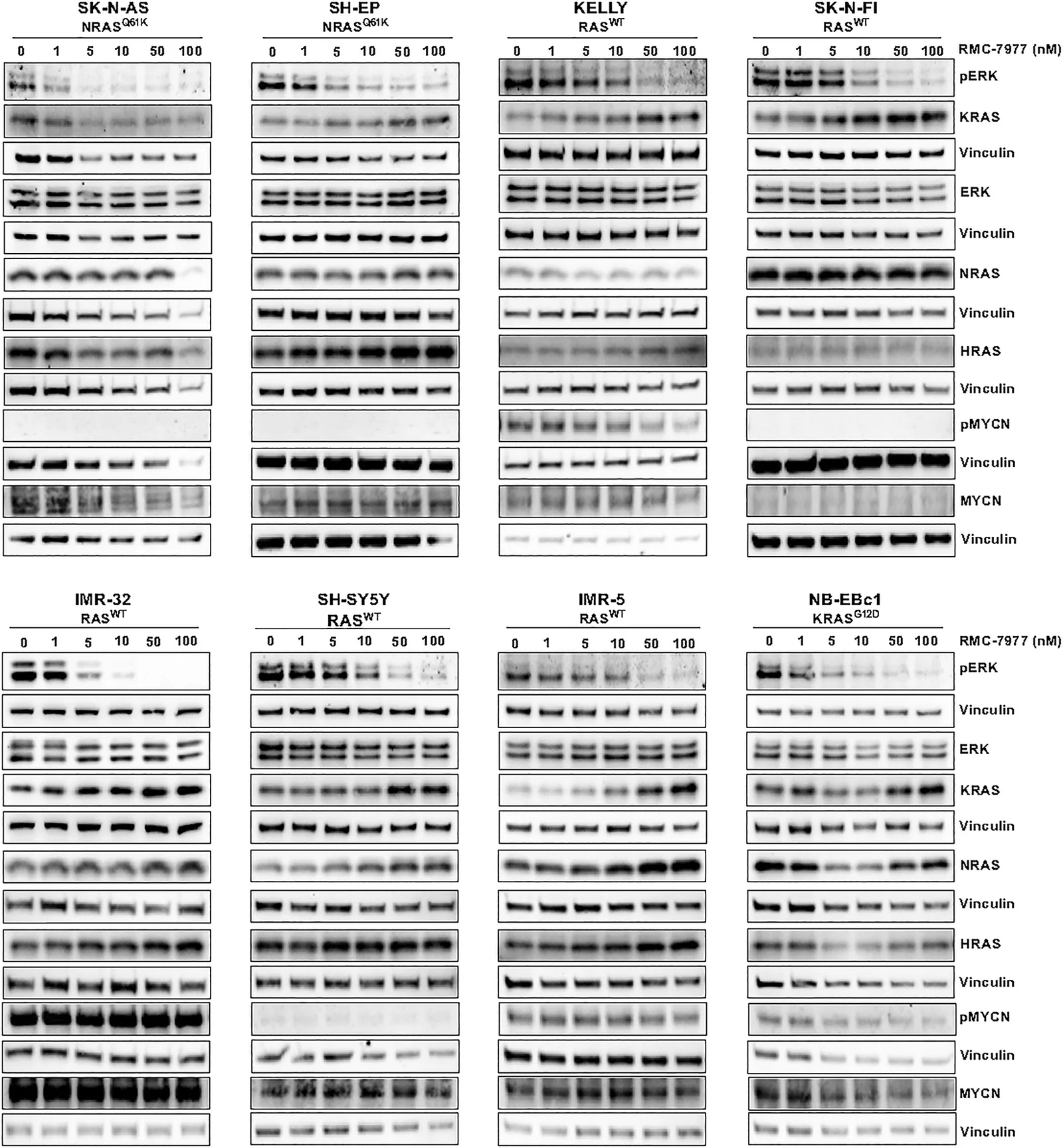
RMC-7977 reduces ERK activation in NBLs. Cells were treated with the indicated concentrations of RMC-7977 or DMSO control and then assessed for effects on ERK activation as described in Fig. 1. Vinculin is shown as a control for loading. The results are representative of three independent experiments for each line.

**Fig. 5:**
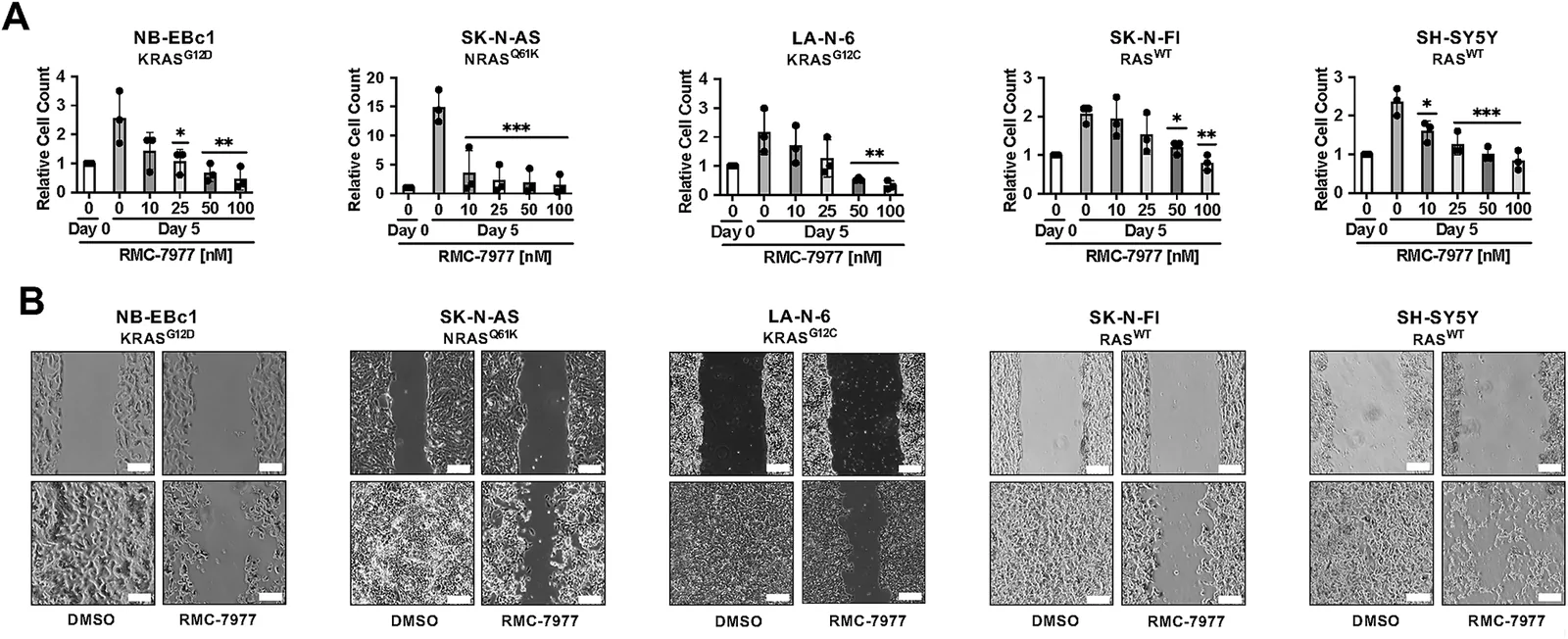
RMC-7977 inhibits the growth and migration of NBL cells in vitro. **(A)** Proliferation of RAS mutant (NB-EBc1, SK-N-AS and LA-N-6) and WT NBLs (SK-N-FI and SH-SY5Y). Results are the average of 3 biological replicates ± SEM shown by bars. p values were determined by comparing values at Day 5 without RMC-7977 (0 nM) to values at different RMC-7977 concentrations using one-way ANOVA. *, *p* < 0.05, **, *p* < 0.01, ***, *p* < 0.001, samples without asterisks were not statistically different from control. **(B)** RMC-7977 inhibits the migration of WT and RAS mutant NBLs. Cells were treated with 50 nM RMC-7977 and subjected to a scratch assay. White scale bars, 500 μm. Quantification of these data is shown in Supplemental Fig. 10A.

During the course of our studies, Holderfield and colleagues described a pan-RAS(ON) pharmacological inhibitor (RMC-7977) that targets the GTP-bound state of all RAS isoforms and interferes with effector engagement^30^. Thus, we examined the efficacy of RMC-7977 at inhibiting NBL lines with and without oncogenic RAS mutations. RMC-7977 treatment led to a sustained and concentration-dependent inhibition of ERK-MAPK activation within 1 h of treatment across most NBL lines examined irrespective of RAS mutational status (Fig. 4, Supplementary Figs. 7A-B, 8 & 9). CHP-134 cells, however, were refractory to RAS inhibition with RMC-7977 (Supplementary Fig. 7C). The effects of RMC-7977 on pMYCN, MYCN, pMEK, MEK, CYPA, DUSP6, and RAS isoforms levels varied between the NBL lines (Fig. 4, Supplementary Fig. 9). Furthermore, the inhibitory effect of RMC-7977 on signaling did not necessarily translate to a biological effect as only a subset of NBL lines (NB-EBc1, SK-N-AS, LA-N-6, SK-N-FI, and SH-SY5Y) showed a reduction in cell proliferation, cell migration, and/or anchorage-independent growth (Figs. 5A-B and Supplementary Figs. 10A-B). For example, RMC-7977 treatment decreased pERK levels in IMR-5, IMR-32, and SH-EP cells (Fig. 4, Supplementary Figs. 10–9) but did not affect proliferation or anchorage-independent growth of these cells (Supplementary Figs. 9C & D). Thus, these findings reveal that WT RAS plays a critical role in sustaining oncogenic signaling in a subset of NBLs lacking RAS mutations.

**Fig. 6:**
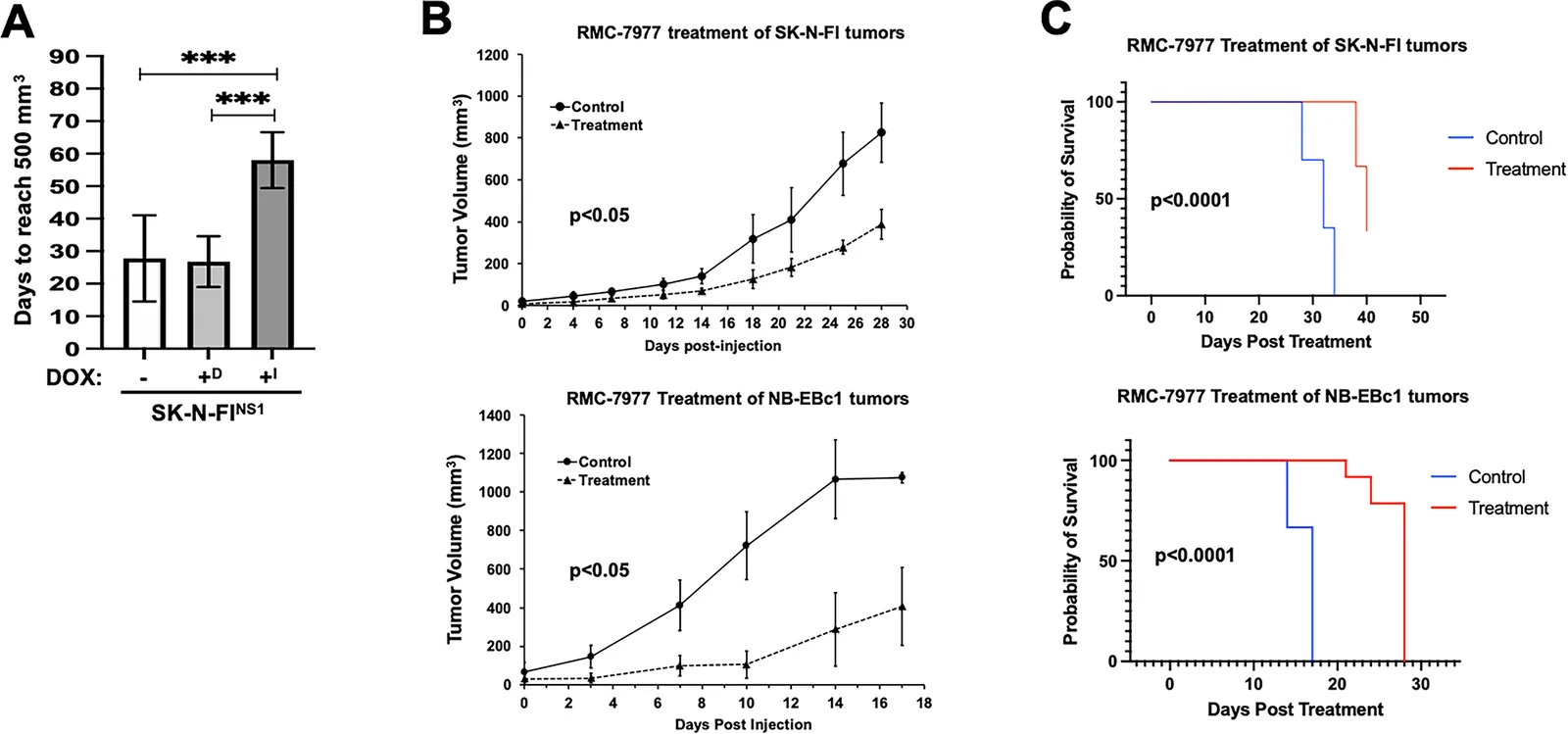
Inhibition of RAS reduces NBL orthotopic tumors. **A)** SK-N-FI^NS1^ were injected orthotopically into the adrenal glands of athymic nude mice and then treated with or without DOX the following day (+^I^) or once tumors reach 50-100mm^3^ (+^D^). Tumor volume was quantified by ultrasound. *n* = 4 for each condition. **(B)** Same as in **(A)** except mice were injected with SK-N-FI or NB-EBc1 cells then treated with either vehicle or RMC-7977 by oral gavage once tumors were detected by ultrasound. *n* = 4 for each condition. The differences in tumor growth were statistically significant with *p* < 0.05. **(C)** Kaplan-Meier survival curves were compared between RMC-7977 treated group and vehicle using Log rank (Mantel-Cox) test. The RMC-7977 treated mice had statistically longer survival than those treated with the vehicle (*p* < 0.0001).

Next, we tested the dependence of NBL cells on RAS for their tumor forming capacity. DOX-induced expression of NS1 reduced the tumorigenic growth of SK-N-FI but not KELLY cells (Fig. 6A and Supplementary Fig. 11). However, this inhibition was only seen when NS1 was induced immediately following cell implantation. Similarly, RMC-7977 reduced the tumorigenic activity of both SK-N-FI and NB-EBc1 cells (Fig. 6B) and increased the survival of mice bearing either NBL line, nearly doubling the survival of NB-EBc1 injected animals (Fig. 6C). These results further support the conclusion that inhibition of RAS activity reduces tumor growth in a subset of NBL tumors, even in the absence of oncogenic RAS mutations.

## Discussion

Despite the paucity of RAS mutations in NBL, the potential of RAS as a therapeutic target in this disease has gained increased interest^49^. Indeed, MEK-ERK pathway inhibition reduces the tumorigenic growth of relapsed NBLs containing mutations in the RAS pathway^25,50^. Our data highlight the efficacy of direct RAS inhibition in RAS-mutant NBLs. Using a combination of newly described RAS pharmacological inhibitors, we demonstrate that this subset of NBLs is dependent on mutant RAS for ERK-MAPK activation, proliferation, migration, and anchorage-independent growth. While these mutations likely arose during chemotherapy^25^, our data suggest that the presence of mutant *RAS* alleles may confer a therapeutic vulnerability in these tumor cells. In agreement with these findings, similar results were recently described with LA-N-6^51^. Thus, pharmacological inhibition of oncogenic RAS may present a new clinical approach in treating a subset of RAS-mutant NBLs, thereby expanding the potential therapeutic landscape for this disease following failure of first line therapies.

While RAS mutations are rare in primary NBL, several upstream regulators of RAS are dysregulated in this disease including ALK, PTPN11, NF1, and GAB2^12,26–28,52^ which may lead to enhanced RAS activity and thus a potential role for WT RAS in NBL^12,26–28,52^. Loss of NF1 results in elevated RAS GTP levels^28,52^ and indeed, we observed elevated RAS GTP in SK-N-FI cells. However, SH-EP cells also exhibited high RAS GTP levels although the levels of NF1 in these cells appear to be relatively normal^52^. This discrepancy may be due to the presence of an activated NRAS allele in SH-EP cells. We observed that pharmacological inhibition of WT RAS with the panRAS(ON) inhibitor, RMC-7977, decreased oncogenic signaling, i.e., reduced ERK-MAPK activation, in most NBL lines examined, including NBLs lacking RAS mutations. Similarly, our RAS inhibitory monobodies reduced ERK-MAPK activation, albeit in a more restricted subset of NBLs. This difference was likely due to several factors. First, the restricted specificity of the RAS monobodies (NS1 inhibits only HRAS and KRAS4B; Mb24 inhibits only NRAS) did not allow for the simultaneous inhibition of all RAS isoforms as is possible with RMC-7977. Indeed, our data indicate that NBLs express abundant KRAS4B and NRAS isoforms, which are refractory to Mb24 and NS1 inhibition, respectively. Second, targeted inhibition of RAS in many cases resulted in a compensatory increase in RAS isoform expression. For example, inhibition of HRAS and KRAS4B with NS1 in SK-N-FI cells increased expression of both H/KRAS isoforms. Furthermore, pharmacological inhibition of RAS led to increased RAS levels in many of the NBLs lines examined. However, in these cases, the increase in RAS isoforms was not sufficient to alleviate the sustained inhibition of ERK over the time course of treatment. Nevertheless, resistance to targeted RAS inhibition is likely to develop in NBLs as has been observed in treatment of other tumors with these inhibitors^53^. These resistance mechanisms may include overexpression and/or mutation of RAS, mutation in downstream RAS pathway components such as RAF, MEK, or MAPK as seen in resistance to chemotherapeutics^54^, activation of RTK pathways such as AXL^55^, or potentially heretofore novel pathways distinct from those observed thus far in adult cancers. Future studies will be needed to define whether resistance to RASi in NBLs, or potentially other pediatric malignancies, differs from resistance mechanisms operative in adult solid tumors.

Although RAS activity was important in driving ERK-MAPK activation in most NBLs examined, this did not necessarily translate into a biological consequence. For example, only a subset of NBL lines were impaired in their proliferative capacity upon RAS inhibition (NB-EBc1, SK-N-AS, SK-N-FI, SH-SY5Y, and LA-N-6) whereas other NBLs were unaffected (CHP-134, IMR-5, IMR-32, and SH-EP) despite the strong inhibitory effects of RMC-7977 on ERK-MAPK pathway activation. This variation in responses to RAS inhibition may be linked to the differential expression of NF1 tumor suppressor which functions as a RAS GTPase activating protein (GAP)^28^. Previous studies revealed a distinct pattern of NF1 expression across both MYCN + and MYCN- NBL cells^28,52^. Indeed, KELLY, CHP-134, SH-SY5Y, and IMR-32 displayed high NF1 expression while SK-N-AS, NB-EBc1, SK-N-FI and CHP-134 had low or undetectable levels (Supplementary Table 1). Both SK-N-FI and NB-EBc1 cells exhibited high basal RAS GTP levels and were sensitive to RAS inhibition. However, we did not observe a clear correlation between NF1 levels, RAS GTP loading, and sensitivity to RAS inhibitors. CHP-134, SH-EP, and SH-SY5Y cells exhibited elevated RAS GTP levels, yet were not dependent on RAS for their proliferation or anchorage-independent growth. These results suggest that other RAS independent signaling pathways play a more prominent role in the tumorigenic properties of these NBLs. Finally, although RMC-7977 reduced growth of SK-N-FI and NB-EBc1 tumors and prolonged the time to reaching humane endpoints, we did not observe tumor regression. However, these experiments do not account for potential role of the immune system in enhancing the anti-tumor effects of RAS inhibitors^56^.

While SK-N-FI cells were sensitive to NS1-mediated inhibition of RAS both in vitro and in vivo, NB-EBc1, KELLY and IMR-5 cells were refractory to NS1’s effects. In the case of NB-EBc1, this was due in part to insufficient expression of the monobody to effectively inhibit RAS nanoclustering^57^. In addition, the restricted specificity of our monobodies (NS1 inhibition of HRAS and KRAS4B; Mb24 inhibition of NRAS) did not allow for the simultaneous inhibition of all four RAS isoforms as seen with RMC-7977. For example, NS1 was expressed at higher levels in KELLY vs. SK-N-FI cells, yet it did not impact ERK-MAPK activation or tumor formation of KELLY cells (Supplementary Figs. 5A & 11). These results lead to several conclusions. First, SK-N-FI cells are reliant on KRAS since the level of NRAS and HRAS in these cells is not sufficient to overcome the inhibition of KRAS by NS1. Second, KELLY and SK-N-FI are dependent on RAS activity for ERK-MAPK activation based on their sensitivity to RMC-7977. Third, it is likely that other RAS isoforms, i.e., NRAS, contribute to the tumorigenic activity of KELLY cells given the restricted specificity of NS1 to HRAS and KRAS4B [this study and^29^ and its inability to impact the growth of KELLY tumors.

### Limitations of current study

Although our findings demonstrate the importance of RAS activity in a subset of RAS mutant as well as RAS WT NBLs and strengthen the case for targeted RAS inhibition as a potential therapeutic strategy for NBL treatment, further research will be needed to better define the molecular subtypes of NBLs that will benefit from RAS-targeted therapies. While chemotherapy may drive RAS mutation, targeted inhibition of RAS may prove beneficial as a second line therapy, albeit in a low percentage of patients (Supplementary Table 2). However, RAS inhibition alone is not likely to be curative, as we only observed a reduction in tumor growth rather than tumor regression (Fig. 6). Thus, targeting RAS in combination with standard of care therapy may prove more effective. Nevertheless, targeted inhibition of RAS in a subset of NBL patients may lead to an objective anti-tumor response as illustrated by results with SK-N-FI and NB-EBc1. A future challenge will be to define biomarkers to stratify NBLs for treatment with RASi’s. While low NMYC/cMYC may be biomarkers for the anti-proliferative response to RASi’s, SH-EP and SK-N-AS do not fit that prediction. SK-N-AS has high cMYC levels yet responds whereas SH-EP has low NMYC/cMYC levels and does not response to RASi’s. Thus, while MYC levels may be predictive, there remain additional factors that likely contribute to the anti-proliferative effects of RASi’s in NBLs. The presence of a RAS mutation and loss of NF1 appear to be most predictive of a response to RASi’s. In the case of WT RAS NBLs, however, future work will be needed to identify additional RASi-responsive lines and define biomarkers predicative of such an anti-tumor response. Finally, given the lower mutational burden of pediatric cancers vs. adult tumors^58^, NBLs may exhibit a distinct spectrum of resistance mechanisms in response to RASi’s compared to adult solid tumors. It will be critical to define such mechanisms that will inevitably develop in NBLs in response to anti-RAS therapeutics as has been observed in treatment of RAS-mutant adult cancers^56^.

## Supplementary Information

Below is the link to the electronic supplementary material.

Supplementary Material 1

## Data Availability

The data generated in this study are available upon request from the corresponding author. The mass spec data on RAS isoforms in neuroblastoma cells has been deposited at PASSEL (https://peptideatlas.org/passel/) with the dataset identifier PASS05928.

## Declarations

### Data and materials availability

Data and materials are available upon request from the authors.
