# Supplementary material for: RAS inhibition limits oncogenic signaling and tumorigenesis in neuroblastoma regardless of RAS mutational status
Source: Sci Rep. 2026 Jul 31;16:35338. doi: 10.1038/s41598-026-63488-x (PMC13719498; doi:10.1038/s41598-026-63488-x)
Supplement: Supplementary file 1 [file 41598_2026_63488_MOESM1_ESM.pdf]

Supplementary Figure 1

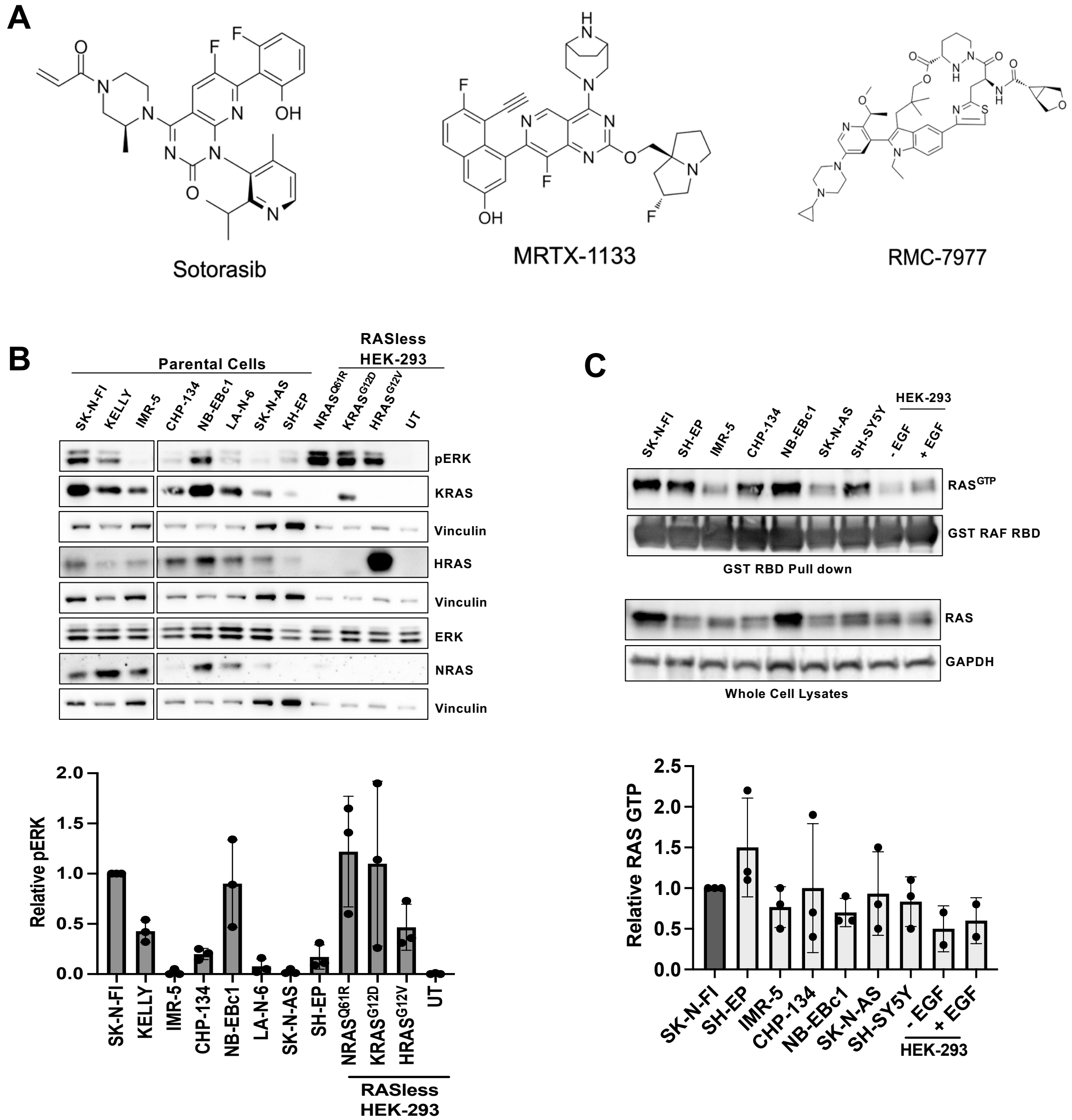

Supplementary Figure 1. RAS inhibitors and basal levels of different RAS isoforms and RAS GTP. A) Structures RAS pharmacological inhibitors used in the study. Sotorasib is a covalent inhibitor specific to KRAS^{G12C}. MRTX-1133 is a non-covalent inhibitor specific for KRAS^{G12D}. RMC-7977 is a panRAS(ON) inhibitor that targets all three RAS isoforms regardless of mutation. **B)** Western blot analysis of pERK, KRAS, NRAS, and HRAS expression in NBL lines. The last four lanes on the right represent lysates from RASless HEK cells expressing NRAS^{Q61R}, KRAS^{G12D}, HRAS^{G12V}, or parental untransfected cells (-). Vinculin was used as a normalization loading control for the samples shown above each blot. Quantification of ERK activation is given below panel. Relative pERK levels represent the pERK/ERK signals for each sample compared to SK-N-FI which was set equal to 1. Results are the average of three independent experiments +/-standard deviation. **C)** RAS activation was measured in lysates of NBL cells using the RAF-RBD pull down assay as described in Materials and Methods. Quantification of three independent assays is shown below the Western blot except for HEK samples which are only n=2. Error bars represent standard deviation. HEK293 cells treated +/-EGF (100 ng/ml for 5 min) were used as a normal reference for RAS GTP levels.

Supplementary Figure 2

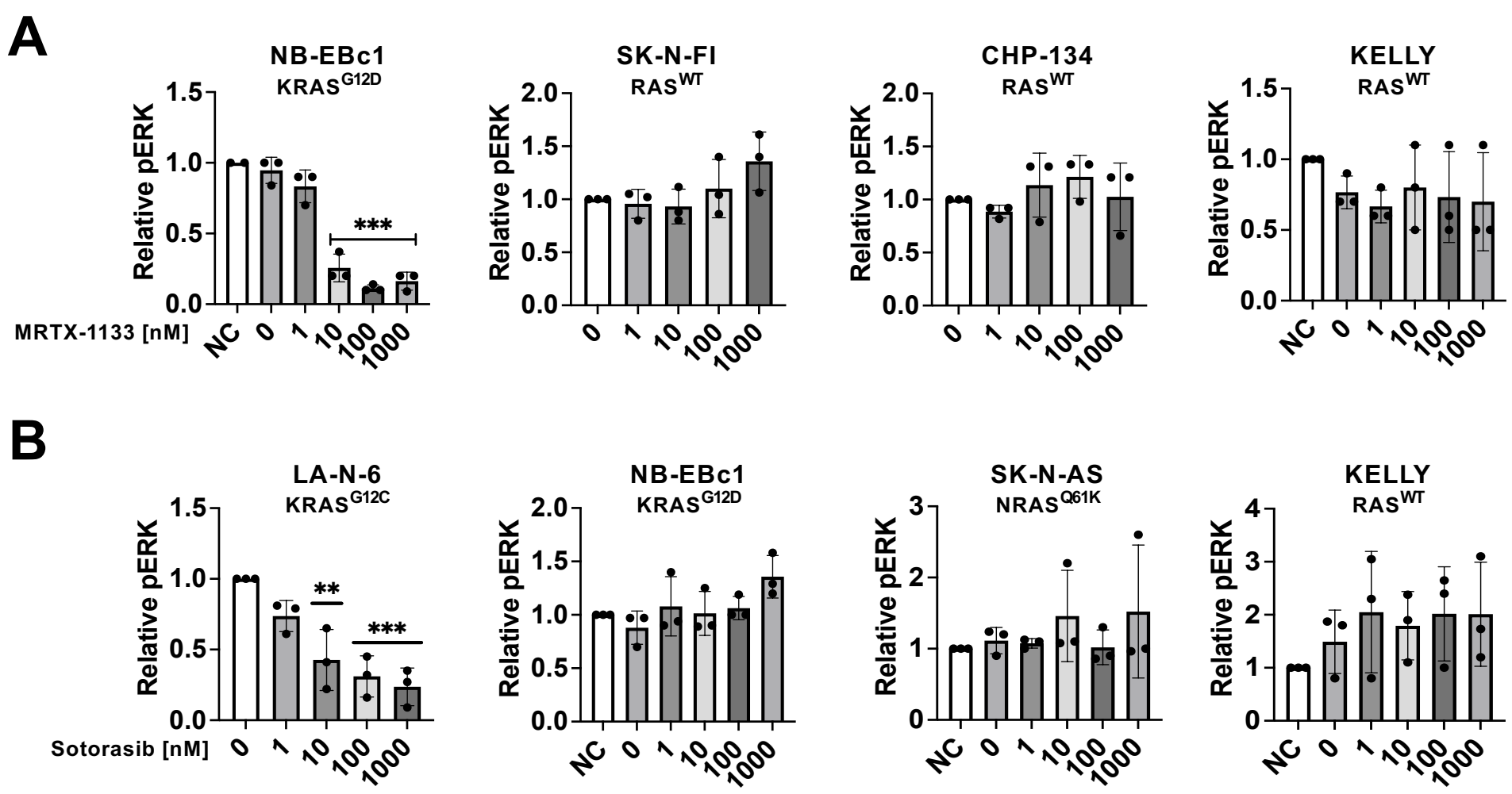

Supplementary Figure 2. Mutation-specific KRAS inhibitors reduce ERK activation in KRAS mutant NBLs. Quantification of pERK activation in Fig. 1. NBL cell lines were treated with the indicated concentrations of MRTX-1133 (A) or Sotorasib (B) for 48 hr respectively. Results are the average of 3 independent experiments. *, $p < 0.05$, **, $p < 0.01$, ***, $p < 0.001$, samples without asterisks were not statistically different.

Supplementary Figure 3

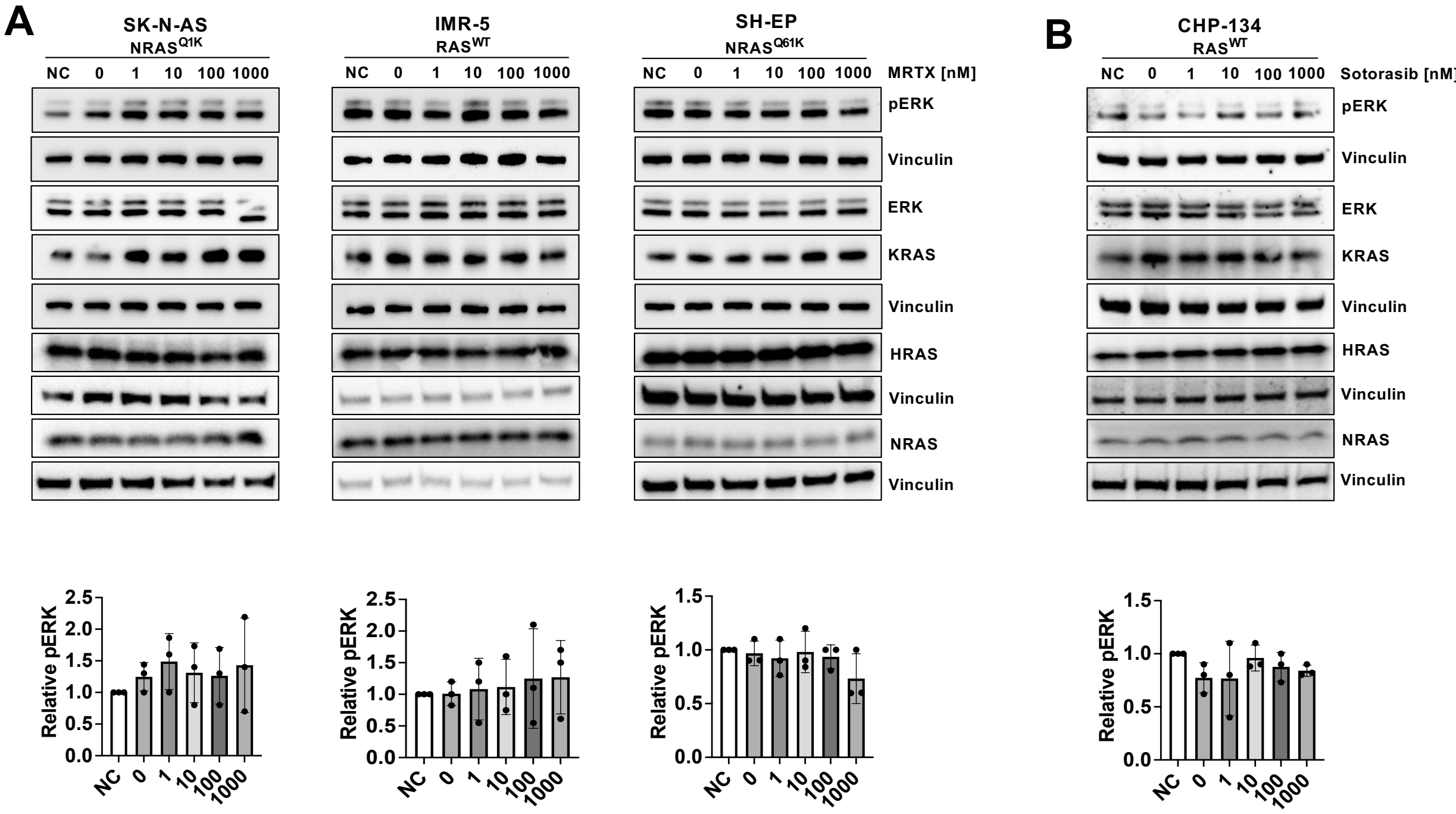

Supplementary Figure 3. Effects of mutation-selective KRAS inhibitors on ERK activation in NBLs. Western blot analysis of pERK, and KRAS expression in NBL lines treated with MRTX-1133 (A) and Sotorasib (B). Vinculin was used as a normalization loading control. Quantification of ERK activation is shown with bar graphs below respective Western blot panels. Results are the average of 3 independent experiments +/-standard deviation. Differences between samples were not statistically significant compared to control as determined by one-way ANOVA.

Supplementary Figure 4

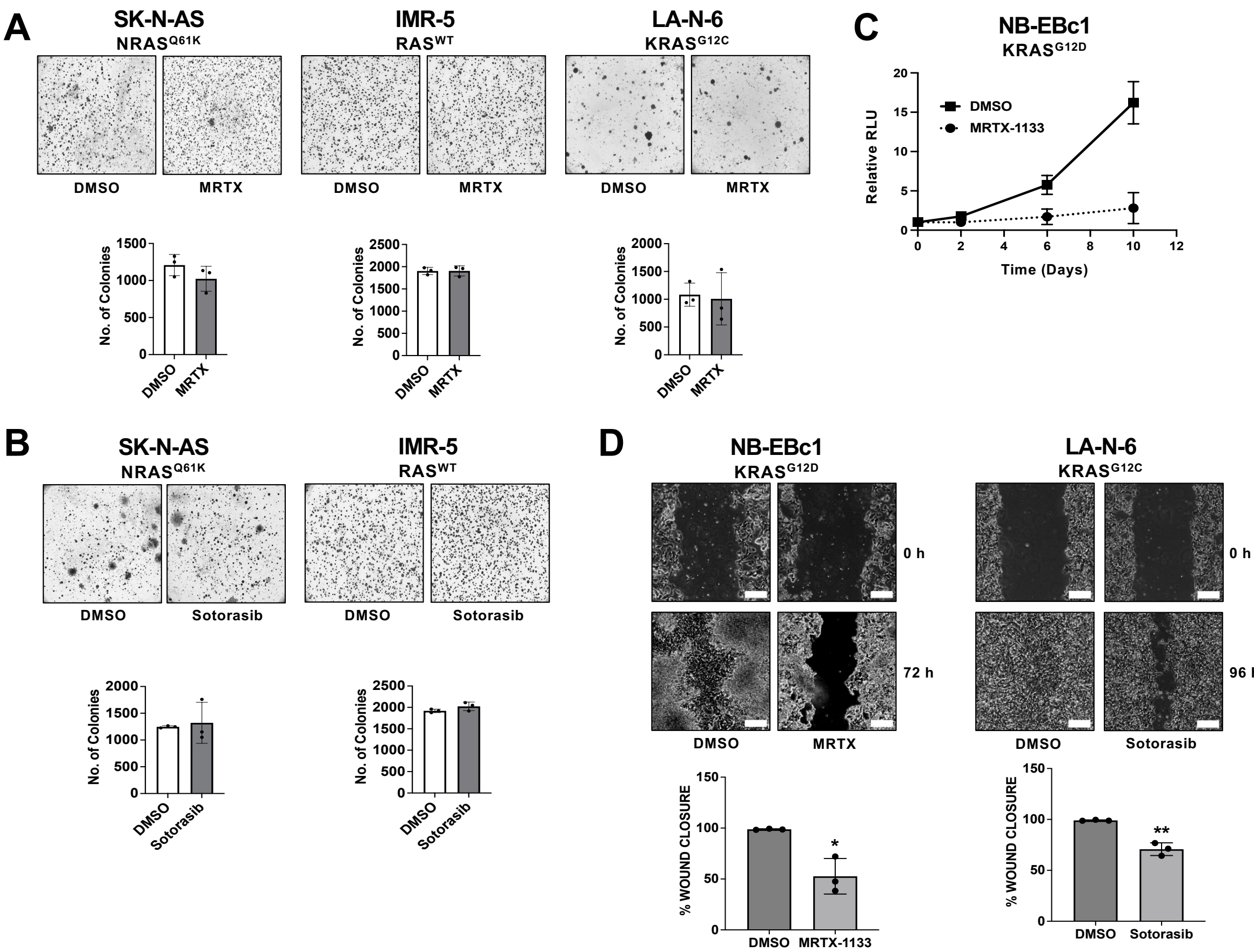

Supplementary Figure 4. Biological effects of mutant selective KRAS inhibitors on NBL cells. A) Anchorage-independent growth of NBL cells treated with MRTX-1133. Shown are representative images of colonies. Quantification of colony number is shown below images. Results are the average colony numbers of three different wells and differences were not statistically significant. B) Same as in (A) except cells were treated with Sotorasib. C) Effects of MRTX-1133 on proliferation of NB-EBc1 cells. Cells were treated with 100 nM MRTX-1133 or DMSO control and analyzed by CellTiterGlo. Results are the average of three independent wells of cells. D) Effects of KRASG12C and KRASG12D inhibition on migration of LA-N-6 and NB-EBc1 cells respectively. Representative images of cells from a scratch assay. Images were quantified and % wound closure is shown in graph below the respective panel of images. Asterisks represent p values which were determined by comparison of % wound closure between DMSO and MRTX/Sotorasib conditions. Results are the average of 3 independent experiments and analyzed by Student's t-test. *, $p < 0.05$, **, $p < 0.01$, ***, $p < 0.001$.

Supplementary Figure 5

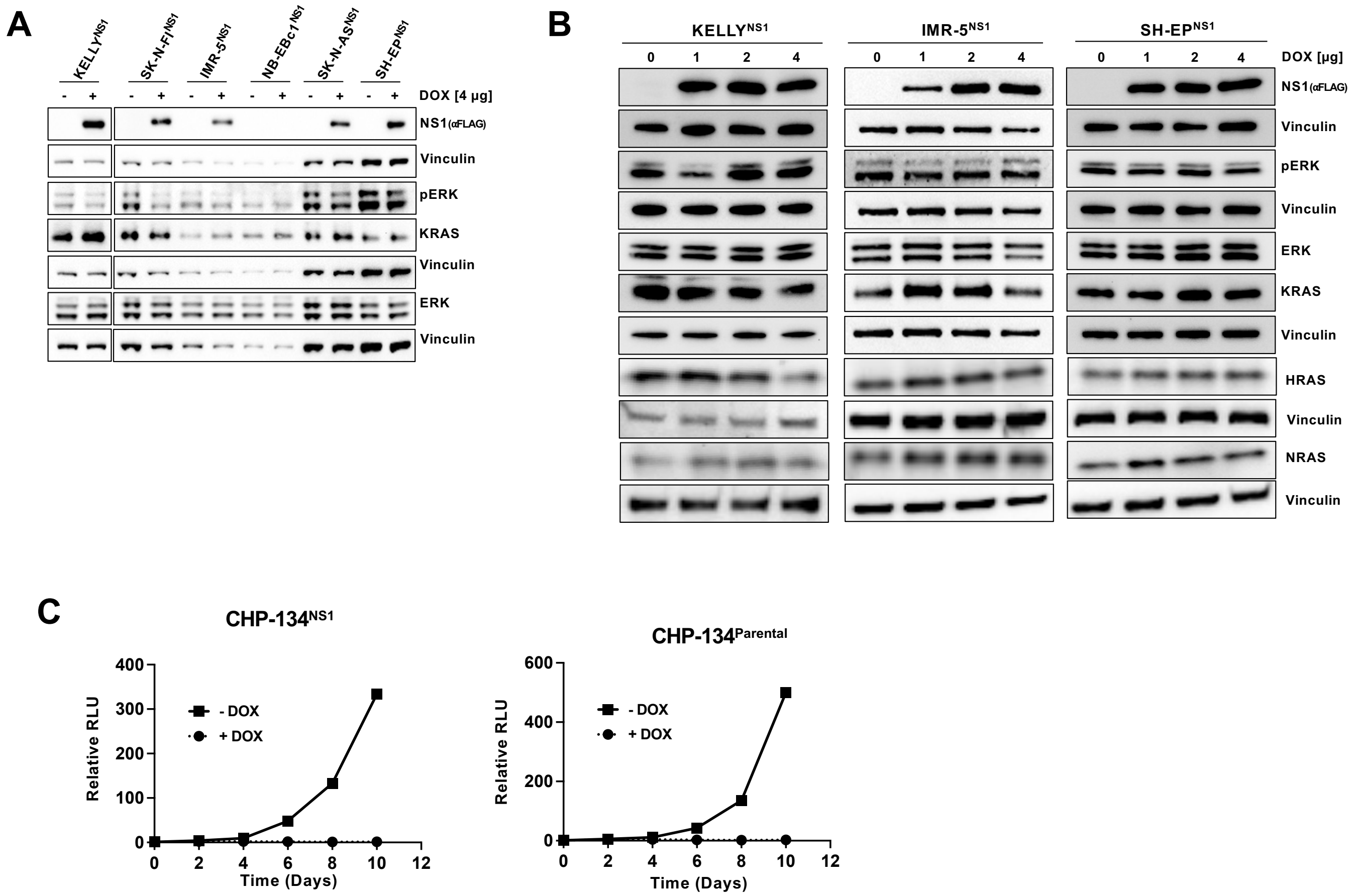

Supplementary Figure 5. Effects of NS1 inhibition of RAS on ERK activation in NBL. A-B) Cells were treated with (+) or without (-) DOX for 2 days and then lysates analyzed with the indicated antibodies. Vinculin was used as a loading control for the samples shown above each blot. B) Effects of NS1 expression on ERK activation. C) Effects of DOX on proliferation of CHP-134^{NS1} and parental CHP-134 cells.

Supplementary Figure 6

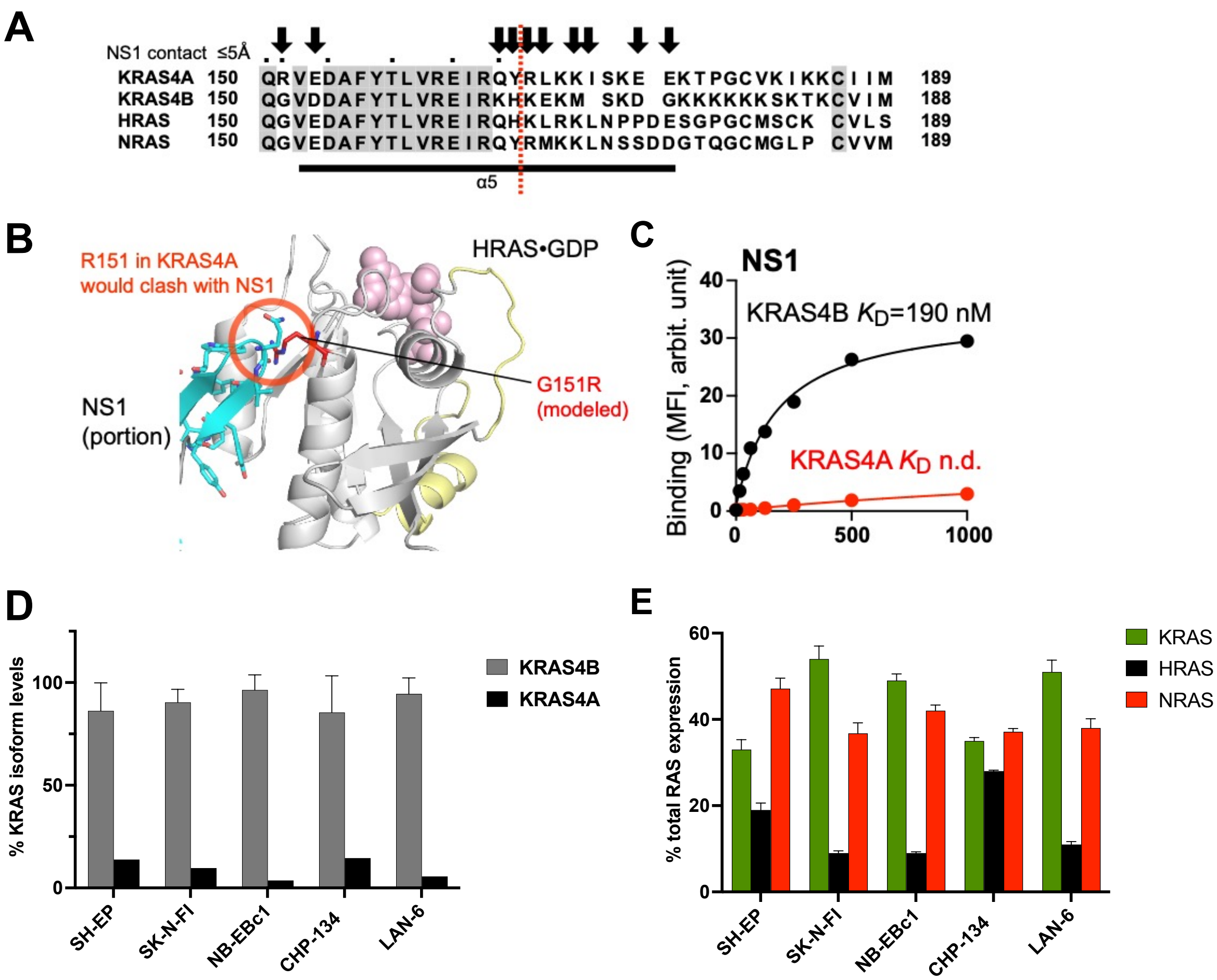

Supplementary Figure 6. Binding specificity of NS1 and RAS isoform abundance in NBL cell lines. A) Sequence alignment of residues 150-189 of RAS isoforms. The arrows indicate HRAS residues that are in contact with NS1 in the crystal structure of HRAS-NS1 complex (29). **B)** The interface between NS1 and HRAS showing potential effect of the G151R change in KRAS4A. **C)** Binding titrations of NS1 displayed on the surface of yeast cells with KRAS4A(GDP) and KRAS4B(GDP). The curve for KRAS4B shows the best fit of the 1:1 binding model. The signals for KRAS4A were too weak to deduce a K_D value. **D)** Relative expression levels of KRAS4B and 4A in NBL lines as determined by quantitative mass spectrometry. n=1 for KRAS4A, n=2 for KRAS4B, ±SEM. **E)** Mean relative abundance of HRAS, KRAS, and NRAS isoforms across NBL lines. N=2 ±SEM.

Supplemental Figure 7

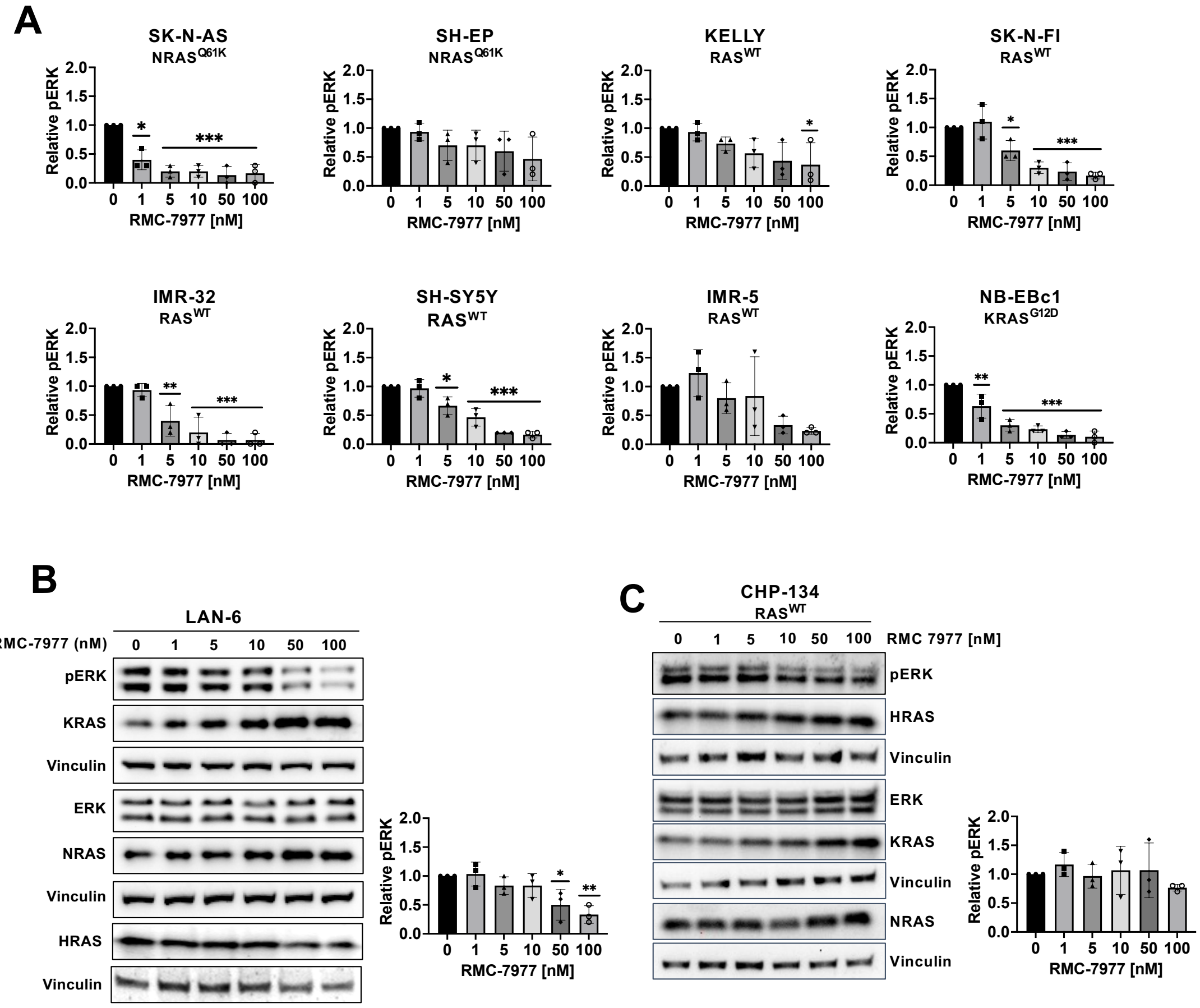

Supplementary Figure 7. RMC-7977 reduces ERK activation in NBLs. (A) Quantification of pERK shown in Fig. 5. Results are the average of 3 biological replicates for each cell line. p values were determined by comparing 0 nM with different concentrations of RMC-7977 using a one-way ANOVA. *, p < 0.05, **, p < 0.01, ***, p< 0.001, samples without asterisks were not statistically different from control. (**B & C)** Effect of RMC-7977 on activation of ERK in LA-N-6 and CHP-134 cells observed in a concentration dependent manner when treated with RMC-7977 for 48 h. Results are an average from 3 biological replicates for each cell line and analyzed as in **(A)**.

Supplementary Figure 8

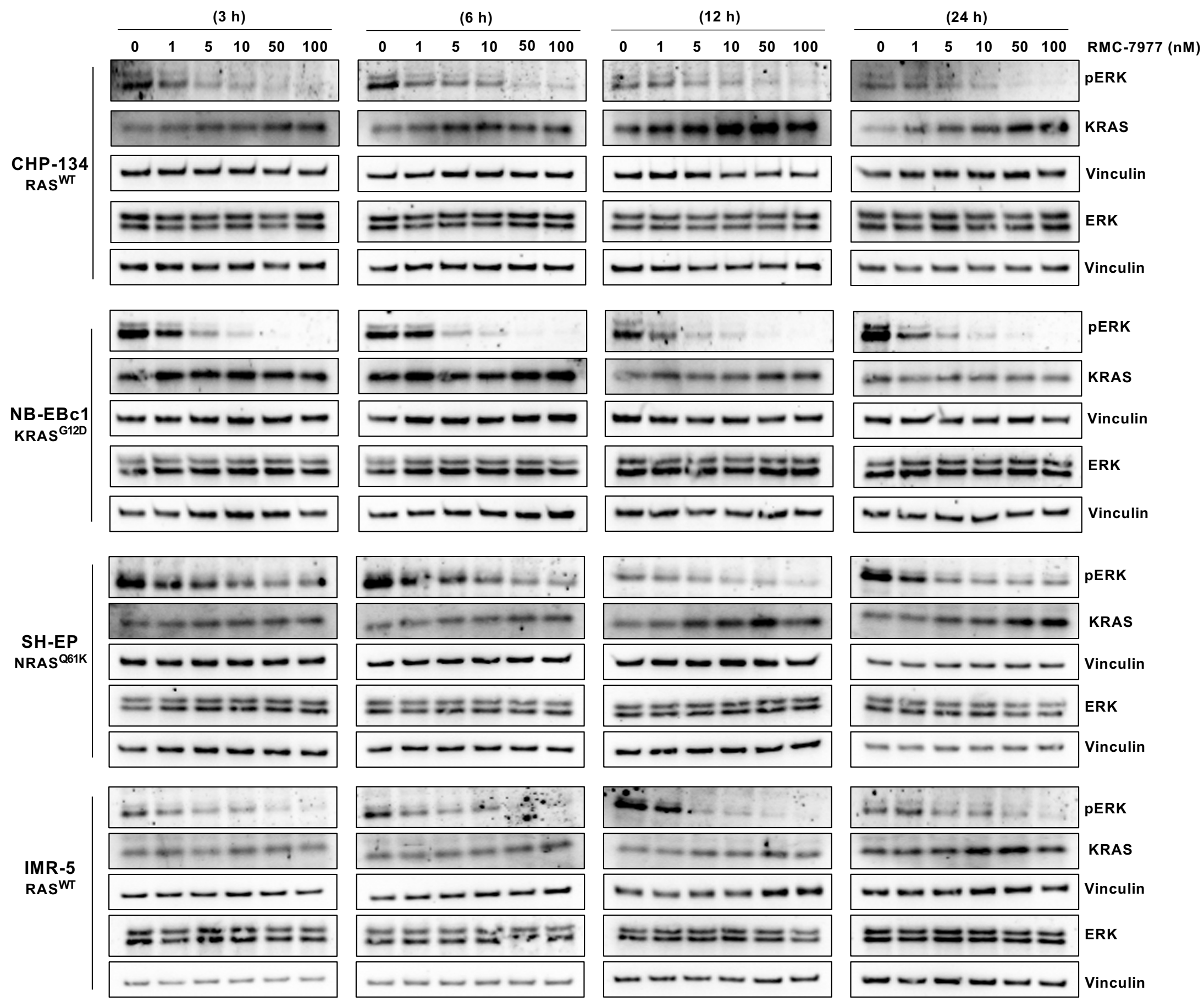

Supplementary Figure 8. Concentration and time-dependent effects of RMC-7977 on ERK activation in NBL cells. NBL lines were treated with the indicated concentrations of RMC-7977 for 3, 6, 12, and 24 hours and then analyzed by Western blot for the activation of ERK. Lysates were also probed for levels of KRAS. Vinculin was used as a loading control for the samples shown above each blot.

Supplementary Figure 9

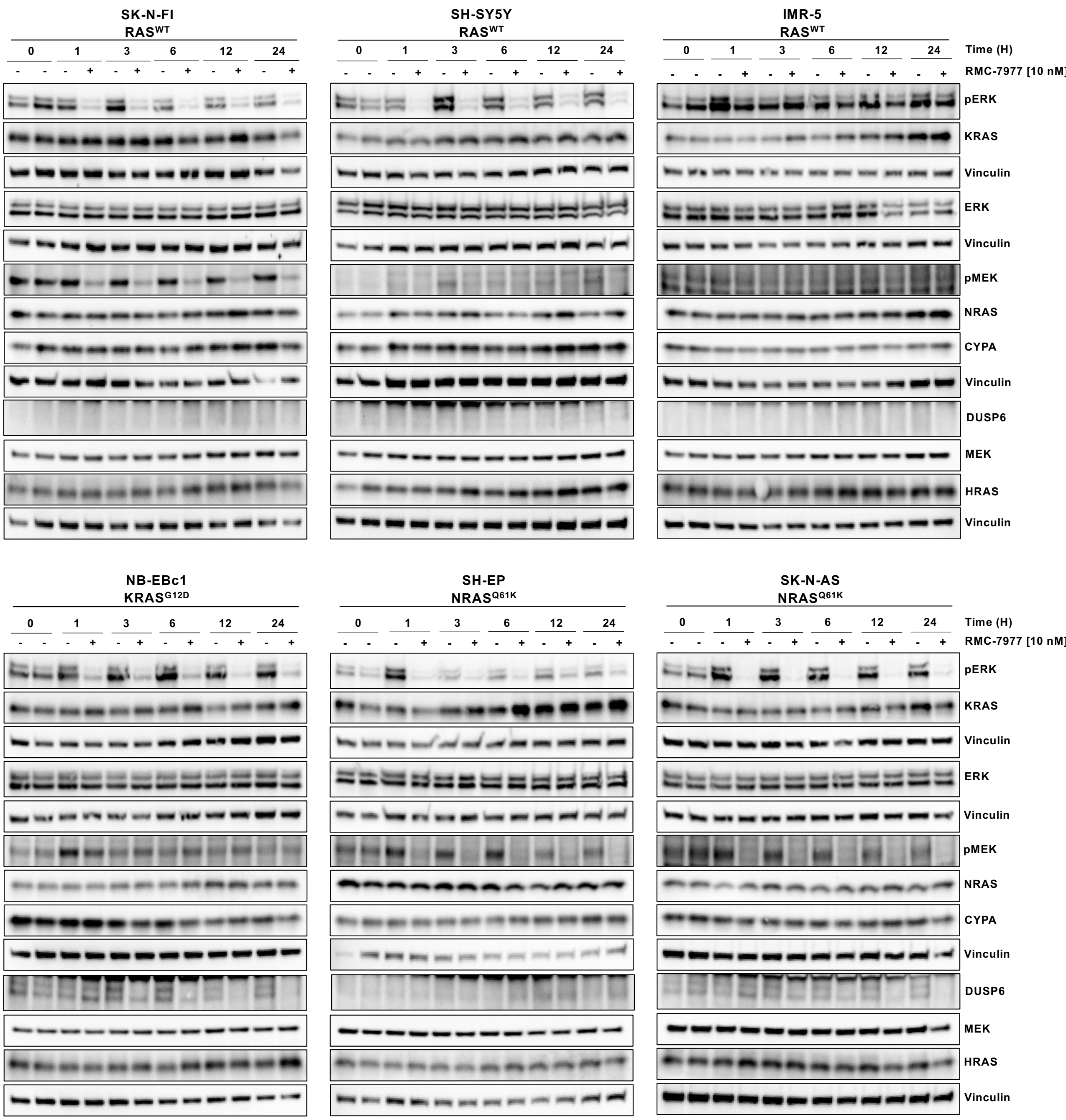

Supplementary Figure 9. Time-dependent effects of RMC-7977 signaling pathways in NBL. NBL lines were treated with 10 nM concentration of RMC-7977 for 0, 1, 3, 6, 12, and 24 hours. Lysates were then analyzed by Western blot with the indicated antibodies to measure effects on MEK-ERK, Cyclophilin A (CYPA), DUSP6 and RAS isoform levels. Vinculin was used as a loading control for the samples shown above each blot.

Supplemental Figure 10

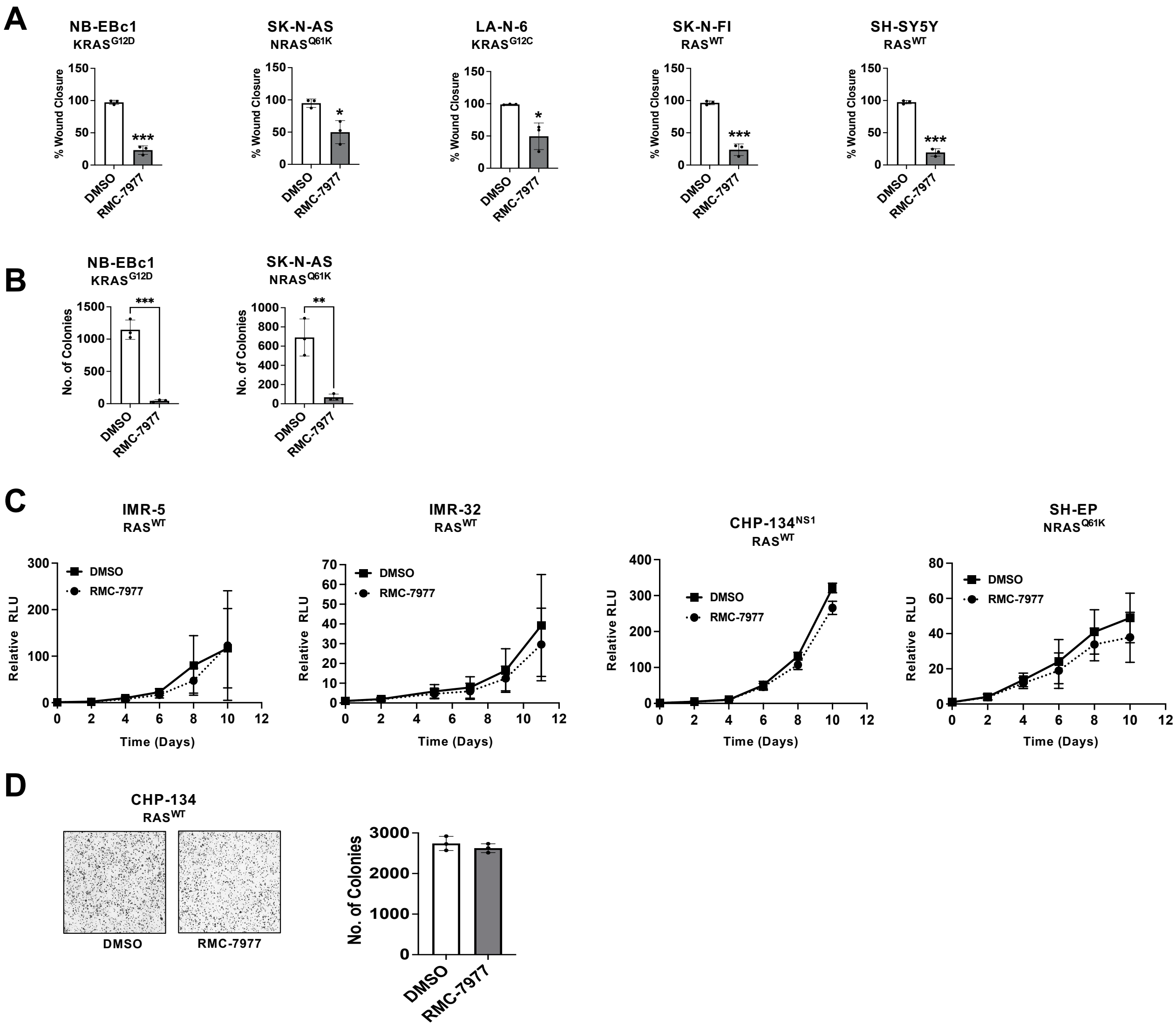

Supplementary Figure 10. RMC-7977 inhibits the growth and migration of NBL cells in vitro. A) Quantification of migration assays from Fig. 5B. Data are average of three biological repeats and quantified with ImageJ as shown in graph. p value was determined by Students t test. *, $p < 0.05$, **, $p < 0.01$, ***, $p < 0.001$, without asterisks were not statistically different from control. **B)** Quantification of anchorage-independent growth assays of NB-EBc1 and SK-N-AS cells treated with RMC-7977. Results are the average of three independent wells. p value was determined by Students t test. **, $p < 0.01$, ***, $p < 0.001$. **C)** Effects of RMC-7977 on proliferation of different NBL cells analyzed by CellTiterGlo for 10 days. **D)** Effects of RMC-7977 on anchorage-independent growth of parental CHP-134 cells. Representative images of colonies are shown. Quantification of colony number is shown below the images.

Supplementary Figure 11

A

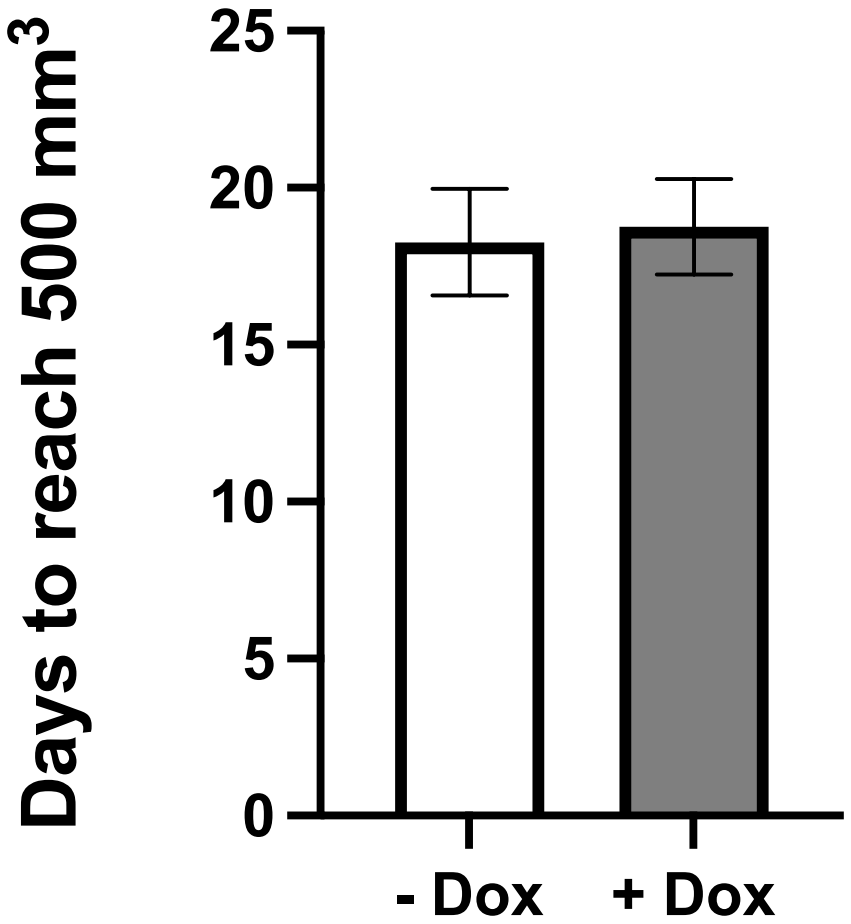

Supplementary Figure 11. Effects of NS1 on NBL tumor growth. A) KELLY^{NS1} cells were injected orthotopically in athymic nude mice and tumor development monitored over time. Induction of NS1 expression with DOX did not affect the growth of tumors.

Supplementary Table 1

Supplementary Table 1: Neuroblastoma cell lines

	SK-N-FI	CHP-134	KELLY	IMR-5	IMR-32	SH-SY5Y	NB-EBc-1	SK-N-AS	SH-EP	LA-N-6
**N-MYC[1]**	WT	AMP	AMP	AMP	AMP	WT	WT	WT	WT	WT
**c-MYC[2]**	Low	Low	Low	Low	Low	Low	Low	High	Low	Low
**ALK**	WT	WT	(F1174L)	WT	WT	(F1174L)	WT	WT	(F1174L)	(D1091N)
**NF1[3]**	-	++	+++	+++	+++	++	+/-	+/-	++	N/A
**PTPN11**	WT	WT	WT	WT	WT	WT	WT	WT	WT	WT
**HRAS**	WT	WT	WT	WT	WT	WT	WT	WT	WT	WT
**NRAS**	WT	WT	WT	WT	WT	WT	WT	(Q61K)	(Q61K)	WT
**KRAS**	WT	WT	WT	WT	WT	WT	(G12D)	WT	WT	(G12C)

[1]Genomic amplification (AMP); [2]mRNA expression; [3]Levels of NF1 as determined by Western blot (see references 28, 50, 52): -, non-detectable; +, low; ++, medium; +++high expression; N/A, not available. WT, wild type.

Supplementary Table 2

Supplementary Table 2. Frequency of RAS mutations in NBL

COSMIC Database[1]						St. Jude Database[5]			
NRAS[2]		KRAS[3]		HRAS[4]		NRAS		KRAS	
Codon	#cases	Codon	#cases	Codon	#cases	Codon	#cases	Codon	#cases
G12D	1	G12V	2	G13R	3	G13R	1	G12D	1
G12R	4	G12A	1	Q61K	6	Q61K	2	G12V	1
A59T	1	G12D	3	Q61R	1	Q61R	1		
Q61K	15	K117N	1						
Q61R	1								
Q61L	1								
Q61E	1								

[1] COSMIC v102, released 21-MAY, 2025; [2] 0.92% of 2613 samples were positive for NRAS mutations; [3] 0.28% of 2512 samples were positive for KRAS mutations; [4] 0.39% of 2537 samples were positive for HRAS mutations. [5] Taken from Brady, S.W., et. al., Nat Comm (2020) https://doi.org/10.1038/s41467-020-18987-4. Aggregate data from 679 NBLs at diagnosis.
